# Aqueous Processed All-Polymer Solar Cells with High Open-Circuit Voltage Based on Low-Cost Thiophene–Quinoxaline Polymers

**DOI:** 10.1021/acsami.3c18994

**Published:** 2024-03-01

**Authors:** Tadele T. Filate, Seungjin Lee, Leandro R. Franco, Qiaonan Chen, Zewdneh Genene, Cleber F. N. Marchiori, Yoonjoo Lee, Moyses Araujo, Wendimagegn Mammo, Han Young Woo, Bumjoon J. Kim, Ergang Wang

**Affiliations:** † Department of Chemistry and Chemical Engineering, Chalmers University of Technology, SE-412 96 Göteborg, Sweden; ‡ Department of Chemistry, Addis Ababa University, P.O. Box 33658, 1000 Addis Ababa, Ethiopia; § Department of Chemical and Biomolecular Engineering, Korea Advanced Institute of Science and Technology (KAIST), 34141 Daejeon, Republic of Korea; ∥ Energy Materials Research Center, Korea Research Institute of Chemical Technology (KRICT), 34114 Daejeon, Republic of Korea; ⊥ Department of Engineering and Physics, Karlstad University, 65188 Karlstad, Sweden; # Department of Chemistry, Korea University, 02841 Seoul, Republic of Korea; ¶ Materials Theory Division, Department of Physics and Astronomy, Uppsala University, 75120 Uppsala, Sweden

**Keywords:** oligo(ethylene glycol), low-cost, aqueous-processable, all-polymer solar cell, eco-compatibility, open-circuit voltage

## Abstract

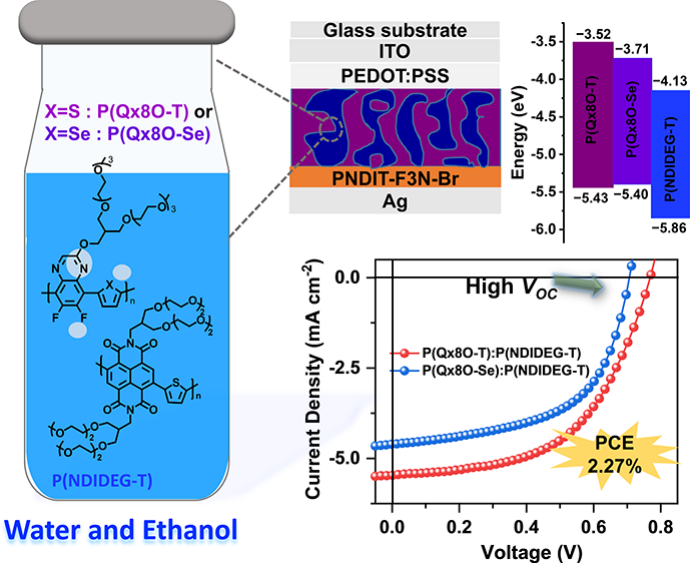

Eco-friendly solution processing and the low-cost synthesis of photoactive materials are important requirements for the commercialization of organic solar cells (OSCs). Although varieties of aqueous-soluble acceptors have been developed, the availability of aqueous-processable polymer donors remains quite limited. In particular, the generally shallow highest occupied molecular orbital (HOMO) energy levels of existing polymer donors limit further increases in the power conversion efficiency (PCE). Here, we design and synthesize two water/alcohol-processable polymer donors, poly[(thiophene-2,5-diyl)-*alt*-(2-((13-(2,5,8,11-tetraoxadodecyl)-2,5,8,11-tetraoxatetradecan-14-yl)oxy)-6,7-difluoroquinoxaline-5,8-diyl)] **(P(Qx8O-T))** and poly[(selenophene-2,5-diyl)-*alt*-(2-((13-(2,5,8,11-tetraoxadodecyl)-2,5,8,11-tetraoxatetradecan-14-yl)oxy)-6,7-difluoroquinoxaline-5,8-diyl)] (**P(Qx8O-Se)**) with oligo(ethylene glycol) (OEG) side chains, having deep HOMO energy levels (∼−5.4 eV). The synthesis of the polymers is achieved in a few synthetic and purification steps at reduced cost. The theoretical calculations uncover that the dielectric environmental variations are responsible for the observed band gap lowering in OEG-based polymers compared to their alkylated counterparts. Notably, the aqueous-processed all-polymer solar cells (aq-APSCs) based on **P(Qx8O-T)** and poly[(*N*,*N*′-bis(3-(2-(2-(2-methoxyethoxy)-ethoxy)ethoxy)-2-((2-(2-(2-methoxyethoxy)ethoxy)ethoxy)-methyl)propyl)naphthalene-1,4,5,8-bis(dicarboximide)-2,6-diyl)-*alt*-(2,5-thiophene)] (**P(NDIDEG-T)**) active layer exhibit a PCE of 2.27% and high open-circuit voltage (*V*_OC_) approaching 0.8 V, which are among the highest values for aq-APSCs reported to date. This study provides important clues for the design of low-cost, aqueous-processable polymer donors and the fabrication of aqueous-processable OSCs with high *V*_OC_.

## 1 Introduction

State-of-the-art organic solar cells (OSCs) with power conversion efficiencies (PCEs) of around 19%^1−8^ continue to attract great attention in academia and the industry due to their potential for real-life applications. However, there remain more challenges in OSC research, such as eco-friendly solution processing and low-cost synthesis of photoactive materials to put OSCs in the forefront of the electronics market.^9,10^ Researchers have strived to make conjugated materials soluble in less toxic solvents (i.e., nonhalogenated solvents including toluene, xylenes, and trimethylbenzenes) for eco-friendly fabrication of OSCs because halogenated solvents pose serious threats to human health and the environment.^9,11−14^ However, there is still a question mark about how “green” these nonhalogenated solvents are since they may still pose serious health hazards and harmful environmental impacts.^15^ Encouragingly, it has been discovered that utilization of ionic pendants or nonionic oligo(ethylene glycol) (OEG) groups in the side chains can enable the processing of conjugated materials in alcohol and aqueous solvents (i.e., water/alcohol mixtures), which are considered the greenest solvents even compared to the nonhalogenated solvents.^16−20^ For example, Woo *et al*.^21^ developed the first OEG-based materials (PPDT2FBT-A donor and fullerene-based acceptors) processable in aqueous solvents, affording OSCs with a power conversion efficiency (PCE) of 0.75%, and they later enhanced the PCE to 2.51% by various structural modifications.^22,23^ Also, Wang *et al*.^24^ used D-OEG polymer donor, with a newly synthesized alcohol processable, boron-containing, nonfullerene small-molecule acceptor and obtained a PCE of 1.03%. Recently, Tan *et al*.^10^ further raised the PCE of aqueous-processable OSCs (aq-OSCs) to 3.03% using a new polymer donor (PFO3) with OEG-containing furan as the polymer backbone.

Although the development of aqueous-soluble conjugated materials has spurred the research on eco-friendly aq-OSCs, there exist hurdles that should be overcome to move to the next stage. For instance, the number of aqueous-soluble donors is highly limited compared to that of aqueous-soluble n-type acceptors including fullerene derivatives and nonfullerene polymers. Only PPDT2FBT-A and the recently reported PFO-based donors have been successfully used to produce aq-OSCs with PCE > 2%.^10,18,23^ Notably, even for these donors, the open-circuit voltage (*V*_OC_) of the corresponding aq-OSCs is relatively low due to their high-lying highest occupied molecular orbital (HOMO) energy levels (∼−5.2 to −5.3 eV) and associated serious charge recombination.

The synthetic cost of the material is another important factor to consider in the effort to commercialize OSCs. Having a lower synthetic complexity with ease of purification will amount to cheaper OSC materials and industrial scalability. However, the synthesis/purification of most aqueous-soluble materials is generally very difficult and time-consuming compared to that of typical OSC materials due to the hydrophilic nature of OEG or ionic side chains.^25^ Therefore, there is a high demand for new aqueous-soluble donors, having down-shifted HOMO energy levels as well as reduced synthetic steps.

In this respect, quinoxaline (Qx)-based polymers can be a model system to benchmark for developing efficient and economical aq-OSCs. For example, Wang *et al*.^26^ developed Qx-thiophene-based polymers (e.g., TQ1) for efficient and low-cost OSCs in 2010. Recently, another simple Qx-thiophene-based polymer, PTQ10, was further developed, which has become one of the best donors in this class of polymers and exhibited a comparable PCE in OSCs with that of the state-of-the-art donor PM6.^1,27^ It is worth noting that PTQ10 has a lower-lying HOMO energy level (∼−5.5 eV) than most polymer donors, such as P3HT (∼−5.1 eV),^28^ PPDT2FBT (∼−5.4 eV),^29^ PTB7-Th (∼−5.2 eV),^30^ and PBDB-T (∼−5.3 eV),^31^ which is beneficial for producing high *V*_OC_. In addition, You *et al*.^32,33^ reduced the cost of synthesizing PTQ10 further by employing the Mitsunobu reaction while maintaining the synthetic simplicity. Thus, we envisioned that employing the Qx-based backbone would provide a great opportunity for the development of new aqueous-soluble polymer donors.

Herein, two Qx-based polymers, poly[(thiophene-2,5-diyl)-*alt*-(2-((13-(2,5,8,11-tetraoxadodecyl)-2,5,8,11-tetraoxatetradecan-14-yl)oxy)-6,7-difluoroquinoxaline-5,8-diyl)] (**P(Qx8O-T)**) and poly[(selenophene-2,5-diyl)-*alt*-(2-((13-(2,5,8,11-tetraoxadodecyl)-2,5,8,11-tetraoxatetradecan-14-yl)oxy)-6,7-difluoroquinoxaline-5,8-diyl)] (**P(Qx8O-Se)**), are developed, in which branched OEG solubilizing groups are grafted onto the conjugated backbone to ensure sufficient solubility in water/ethanol mixtures. The electronic properties and geometric conformations of these polymers are compared theoretically and experimentally with those of their alkoxylated counterparts. Unlike the alkylated polymers, the OEG-based polymers exhibit high-lying HOMO and low-lying lowest unoccupied molecular orbital (LUMO) energy levels. Notably, the preparation of the polymers is completed in five synthetic steps, effectively reducing the overall synthetic cost. In addition, an aqueous-soluble polymer, poly[(*N*,*N*′-bis(3-(2-(2-(2-methoxyethoxy)-ethoxy)ethoxy)-2-((2-(2-(2-methoxyethoxy)ethoxy)ethoxy)-methyl)propyl)naphthalene-1,4,5,8-bis(dicarboximide)-2,6-diyl)-*alt*-(2,5-thiophene)] (**P(NDIDEG-T)**) is selected as an acceptor to construct aqueous-processed all-polymer solar cells (aq-APSCs) with the synthesized donors due to the advantages of APSCs over polymer:fullerene-based OSCs.^34,35^ As a result, the aq-APSCs based on the **P(Qx8O-T)**:**P(NDIDEG-T)** blend achieve a maximum PCE of 2.27% with a high *V*_OC_ of ∼0.8 V due to the deep HOMO level of **P(Qx8O-T)**. This work lays the groundwork for designing inexpensive, aqueous-soluble polymer donors with deep HOMO energy levels for the fabrication of efficient aq-OSCs.

## 2 Results and Discussion

**Figure 1:**
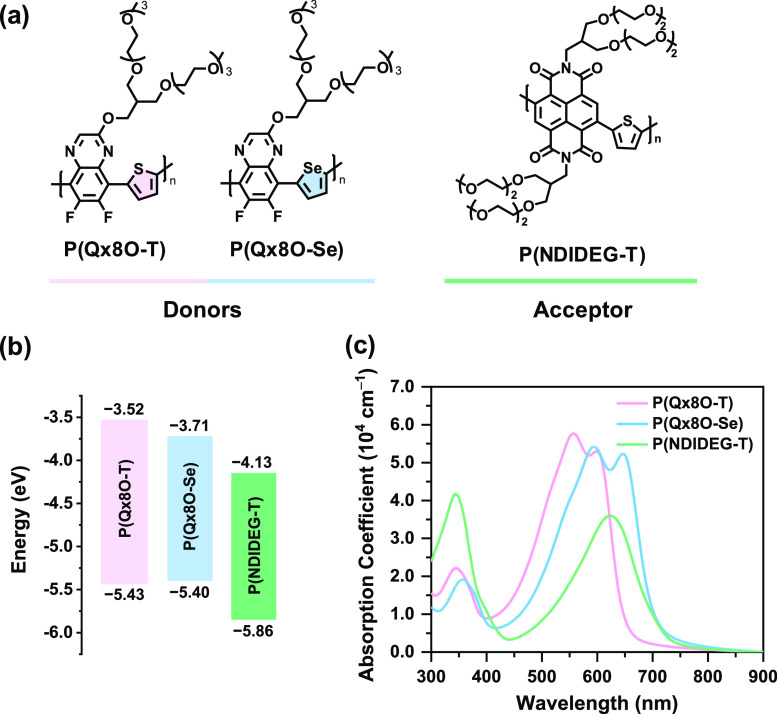
(a) Molecular structures, (b) frontier orbital energy levels of **P(Qx8O-T)**, **P(Qx8-Se)**, and **P(NDIDEG-T)**. (c) Absorption coefficients of the polymer thin films processed from water/ethanol mixtures (15:85 v/v).

The chemical structures of the polymers used in this study are given in Figure 1a. For the **P(Qx8O-T)** and **P(Qx8O-Se)** polymer donors, two different backbones (Qx-thiophene and Qx-selenophene) were employed to modulate their energy levels and intermolecular interactions.^36,37^ It was expected that the sulfur atom in thiophene and the selenium atom in selenophene, which have dissimilar atomic radii and different degree of quinoidal resonance effect,^38^ would result in different molecular packing and electrical properties. Besides, the two fluorine atoms attached to the Qx unit help reduce the HOMO energy levels of both polymers for attaining high *V*_OC_. In addition, the OEG side chains were grafted to the backbone to enable sufficient processability in aqueous solvents. The detailed synthetic procedures of the Qx-based polymers (Scheme S1) are described in the Experimental Section, and their molecular structures were confirmed by proton nuclear magnetic resonance (^1^H NMR) and carbon-13 NMR (^13^C NMR) spectroscopies (Figures S1 and S2, respectively). The number-average molecular weight (*M*_n_) and dispersity (*D̵*) of the polymers are provided in Table 1. **P(NDIDEG-T)** was used as an acceptor because it has good electrical properties and aqueous processability plus well-matched energy levels with the donor polymers.^18^**P(Qx8O-T)** and **P(Qx8O-Se)** showed good solubility in various halogenated and nonhalogenated solvents such as acetone, ethyl acetate, tetrahydrofuran, chloroform, and also in ethanol/water mixtures, ranging from 50 to 95% ethanol (v/v). **P(Qx8O-T)** and **P(Qx8O-Se)** showed solubility of 43.3 and 36.4 mg mL^–1^, respectively, in 85 vol % ethanol. The thermogravimetric analysis (TGA) of **P(Qx8O-T)** and **P(Qx8O-Se)** showed good thermal stability where the decomposition temperatures at 5%-weight loss were 366 and 359 °C, respectively (Figure S3a). No thermal transitions were detected for both polymers from the differential scanning calorimetry (DSC) measurements (Figure S3b).

**Table 1:** Material Properties of **P(Qx8O-T)**, **P(Qx8O-Se)**, and **P(NDIDEG-T)**

polymer	*M*_n_ (*D̵*) [kg mol^–1^]^a^	λ_max_^film^ [nm]^b^	ε_max_^film^ [cm^–1^]^b^	*E*_g_^opt^ [eV]^c^	*E*_HOMO_ [eV]^d^	*E*_LUMO_ [eV]^e^	*E*_g_ [eV]^f^
**P(Qx8O-T)**	16 (2.2)	557, 599	5.8 × 10^4^	1.84	–5.43	–3.52	1.91
**P(Qx8O-Se)**	7.6 (2.2)	594, 647	5.4 × 10^4^	1.67	–5.40	–3.71	1.69
**P(NDIDEG-T)**	26 (3.1)	343, 623	4.2 × 10^4^	1.69	–5.86	–4.13	1.73

a Determined by size-exclusion chromatography using *ortho*-dichlorobenzene as the eluent relative to polystyrene standard.

b Determined from UV–vis absorption spectra of thin films processed from the aqueous solvent.

c Optical band gap calculated from the absorption onset of the thin film.

d *E*_HOMO_ = −(*E*_ox_^onset^ + 5.13) eV.

e *E*_LUMO_ = −(*E*_red_^onset^ + 5.13) eV.

f Electrochemical band gap calculated from the difference of *E*_HOMO_ and *E*_LUMO_.

Notably, in the design of the polymers, their synthetic costs were also considered. We have followed previously reported cost-effective synthetic route^32,39^ where the Qx unit was prepared in three steps from inexpensive 1,4-dibromo-2,3-difluorobenzene. In addition, the synthesis of the intermediates and monomeric molecules included easy purification procedures, with only two column chromatography processes over the five synthetic steps. To calculate the cost, the final amount of each polymer was set to be 1.0 g and computed back to each starting material considering the percentage yield at all stages. The cost of each starting material was taken based on the price quoted in the Sigma-Aldrich catalog for the largest available package. Thus, in this synthetic pathway, the total costs were estimated to be only 44.89 $ g^–1^ for **P(Qx8O-T)** and 55.08 $ g^–1^ for **P(Qx8O-Se)** (Tables S1 and S2), significantly lower than other state-of-the-art polymer donors.^21,32^ In particular, **P(Qx8O-T)** has a comparable low cost as compared to the recently reported aqueous-processable polyfuran homopolymers PFO3 and PFO4.^10^ The low-cost of the polymers makes Qx-based donors promising candidates for the large-scale production of aq-OSCs.

The HOMO and LUMO energy levels (*E*_HOMO_ and *E*_LUMO_) of the polymers were experimentally determined from cyclic voltammetry (Figures 1b and S4, and Table 1). The *E*_HOMO_/*E*_LUMO_ of **P(Qx8O-T)** and **P(Qx8O-Se)** was found to be −5.43/–3.52 and −5.40/–3.71 eV, respectively, from the onset potentials of oxidation/reduction. Notably, both **P(Qx8O-T)** and **P(Qx8O-Se)** showed deeper *E*_HOMO_ values compared to that previously reported for PPDT2FBT-A donor,^18^ and therefore, enhanced *V*_OC_ in the resulting OSCs can be expected (*vide infra*). Replacing thiophene with selenophene in the donor polymer decreases the *E*_LUMO_ and increases the *E*_HOMO_. Consequently, the band gap of **P(Qx8O-Se)** is smaller than **P(Qx8O-T)**, which agrees with previous comparative studies.^37,40^ It is generally believed that selenophene has lower aromatic stabilization energy and can form stronger quinoidal resonance relative to thiophene, which can explain the band gap lowering and slightly upshifted *E*_HOMO_ of selenophene-based polymers, but it has not been clear why they have relatively more downshifted *E*_LUMO_. To gain further insights into this difference between thiophene- and selenophene-based polymers and to understand the experimental results, we conducted DFT-based calculations (detailed are given in the Supporting Information). Figure S5 displays the HOMOs and LUMOs of **P(Qx8O-T)**, **P(Qx8O-Se)**, and **P(NDIDEG-T)** polymers. The HOMO densities of the donor polymers are evenly distributed. However, the HOMO density of the acceptor polymer experiences slight perturbations due to the influence of the thiophene rings, resulting in an asymmetrical distribution along the diimide moieties. In contrast, the LUMO densities of both the donor and acceptor polymers are homogeneously distributed across their respective monomeric units. As a result, this can impact the character of low-lying electronic transitions of the systems upon light absorption. The calculated isotropic polarizability of **P(Qx8O-Se)**, 2060.9 au, is larger than that of **P(Qx8O-T)**, 1927.9 au (Table S3). The stronger electric field created by the larger polarizability of the Se atom can lead to lower *E*_LUMO_. The Se atoms also affect the local electrostatic surface potential (ESP) of the polymer (Figure S6). In the vicinity of the Se-atom in **P(Qx8O-Se)**, the ESP exhibits a relatively more negative appearance than that in the S atom in **P(Qx8O-T)**. A higher molecular polarity index (MPI) of 13.82 for **P(Qx8O-Se)**, compared to 13.35 for **P(Qx8O-T)**, was observed from the integration of the ESP surface. This difference in the MPI values can be attributed to the greater electronegativity of Se relative to S, which is different from the Pauling scale of electronegativity^41^ (refer to the detailed discussion in Note 1 and Figure S7 in the Supporting Information, and Sanderson^42^ and Allred-Rochow scales^43^ ). Here, we conclude that although **P(Qx8O-T)** has a less positive charge as compared to **P(Qx8O-Se)**, the larger ESP and MPI values from the selenophene-based polymer **P(Qx8O-Se)**, resulting from the more electronegative Se atom as compared to the S atom, can explain its downshifted *E*_LUMO_.

Another important aspect here is that the OEG-containing polymer **P(Qx8O-T)** showed significantly different energy levels and band gap compared to the alkylated analog PTQ10 (Table S4 and Figure S8). More specifically, the band gap of **P(Qx8O-T)** was found to be much narrower than that of PTQ10 with up-shifted *E*_HOMO_ and down-shifted *E*_LUMO_. This trend in energy level is commonly observed in the literature for many OEG-containing polymers and their alkylated analogues, but a clear explanation for these differences is still missing.^33,44^ One possible explanation is that the electronegative oxygen atoms in the OEG-based materials draw some electron density through the inductive effect, leading to slightly deeper *E*_LUMO_ but potentially contradicting the destabilization of *E*_HOMO_ in these materials. To further understand this, we optimized the molecular geometry of trimeric donors and acceptor materials with both the OEG and alkyl side chains using DFT calculations at the HSE03/6-311G(d,p) theory level. Figure S9 illustrates the fully relaxed geometries in the ground state, and no visible differences in terms of side-chain orientation or backbone planarity between the OEG-based polymers and their corresponding alkyl-based counterparts are observed. In a vacuum, the OEG-based polymers **P(Qx8O-T)** and **P(Qx8O-Se)** have comparable *E*_LUMO_/*E*_HOMO_ with their alkylated counterparts **P(QxC27-T)** and **P(QxC27-Se)** with minor differences of −0.06/–0.06 and −0.05/–0.03, respectively. To account for the impact of the surrounding environment, the implicit universal solvation model SMD was employed.^45^ We used two solvents, *n*-octanol (ε = 9.86) and 1-fluorooctane (ε = 3.89), to mimic the dielectric constants of the polymeric films as closely as possible. Specifically, *n*-octanol was utilized for OEG-based polymers, while 1-fluorooctane was selected for the alkyl-based polymers. As a result, when OEG is replaced by alkyl side chains, the *E*_LUMO_/*E*_HOMO_ of **P(Qx8O-T)** and **P(Qx8O-Se)** are altered by +0.12/–0.17 eV and +0.14/–0.16 eV, respectively (Figure S10). This is consistent with the experimental results, as illustrated in Figure S10. These findings suggest that the environmental effects may primarily contribute to the observed energy level and band gap differences between OEG-containing and alkylated polymers as an OEG-based polymeric material possesses a higher dielectric constant than that of an alkyl-based polymer.^46^ As mentioned above, the impact of the OEG side chains on the *E*_HOMO_ and *E*_LUMO_ obtained from CV measurements was not well understood previously. The most widely accepted argument was that the enhanced ion permeability of OEG-based polymers results in reduced oxidation and reduction potentials.^47^ This seems plausible since alkylated polymers have hydrophobic side chains that are not favorable to the permeation of ions and polar solvent particles. Hence, the pristine state of the alkylated polymer needs external potential to compel the movement of ions in and out of the thin films.^48^ Conversely, the OEG side chains enhance the interaction of OEG-based polymers with ionic species. In addition to those effects, here, we have shown through our theoretical calculations that the dielectric environment induced by the presence of OEG groups could also influence the *E*_HOMO_ and *E*_LUMO_. Additionally, a significant electrochemical band gap (*E*_LUMO_ – *E*_HOMO_) difference between **P(Qx8O-T)** and **P(Qx8O-Se)** (1.91 eV vs 1.69 eV, Δ*E*_g_ = 0.22 eV) was observed. This difference is larger than the commonly observed band gap differences in alkylated polymers with thiophene–selenophene exchange.^37^ Nevertheless, a similarly significant alteration was observed in OEG-grafted NDI-based polymers with the thiophene–selenophene exchange as compared to their alkylated counterparts.^18^ Therefore, we tentatively attribute this pronounced electrochemical band gap difference to the combined effects of OEG side-chain substitution and thiophene–selenophene exchange.

UV–vis absorption spectra were recorded (Figure 1c) to examine the optical properties of the polymers. The thin films of both **P(Qx8O-T)** and **P(Qx8O-Se)** featured two absorption bands, which are typical characteristics of donor–acceptor copolymers. However, the absorption onset of the **P(Qx8O-Se)** film (738 nm) was red-shifted by 64 nm compared to that of the **P(Qx8O-T)** film (674 nm), mainly due to the larger quinoidal resonance effects and more electron-rich properties of selenophene as compared to thiophene.^49^ This result suggests that the **P(Qx8O-T)**:**P(NDIDEG-T)** blend system would result in better complementary absorption than the **P(Qx8O-Se)**:**P(NDIDEG-T)** pair (Figure 1c). Furthermore, the first electronic transition (S_1_) is the most intense in the visible range and corresponds with the absorption maxima of **P(Qx8O-T)**, **P(Qx8O-Se)**, and **P(NDIDEG-T)** (Figure S11). **P(Qx8O-Se)** exhibits a more red-shifted S_1_ transition (Figure S11), which corresponds well with its lower band gap than **P(Qx8O-T)**. Moreover, at higher energies, a set of weaker electronic transitions account for the lower-intensity absorption bands of the polymers.

To investigate the molecular aggregation in solution, the temperature-dependent aggregation (TDA) properties of the polymers in water/ethanol (15:85 v/v) solution were analyzed by UV–vis spectroscopy (Figure S12). Although both polymers displayed weakening of the main absorption bands as the temperature increased from 20 to 80 °C, their maximum absorption wavelengths (λ_max_s) barely changed, indicating that **P(Qx8O-T)** and **P(Qx8O-Se)** have favorable preaggregation properties in aqueous solution, even at high temperature, which is essential for producing optimal blend morphology in OSCs.^50,51^ For both polymers, the intensity of the shoulder peak generally diminished as the temperature increased from 20 to 80 °C. However, the shoulder peak (*ca*. 650 nm) of **P(Qx8O-Se)** prevailed even at 80 °C. This result suggests that **P(Qx8O-Se)** has a strong aggregation tendency compared to **P(Qx8O-T)**, which could also be confirmed by larger surface roughness in atomic force microscopy (AFM) measurement of the **P(Qx8O-Se)**:**P(NDIDEG-T)** blend, as discussed below. To further understand this, we looked into the simulated conformations (Note 2). The presence of selenium in **P(Qx8O-Se)** induces the planarity of the polymer backbone, whereas twisting was observed in **P(Qx8O-T)**. This is mainly because of the higher quinoidal resonance contribution from the selenophene ring, which is connected to the larger p-orbital size and higher polarizability of the Se atom (see Table S3). Thus, the high aggregation tendency of **P(Qx8O-Se)** might have emanated from its relatively planar conformation, as shown in Figure S9.^52^

**Figure 2:**
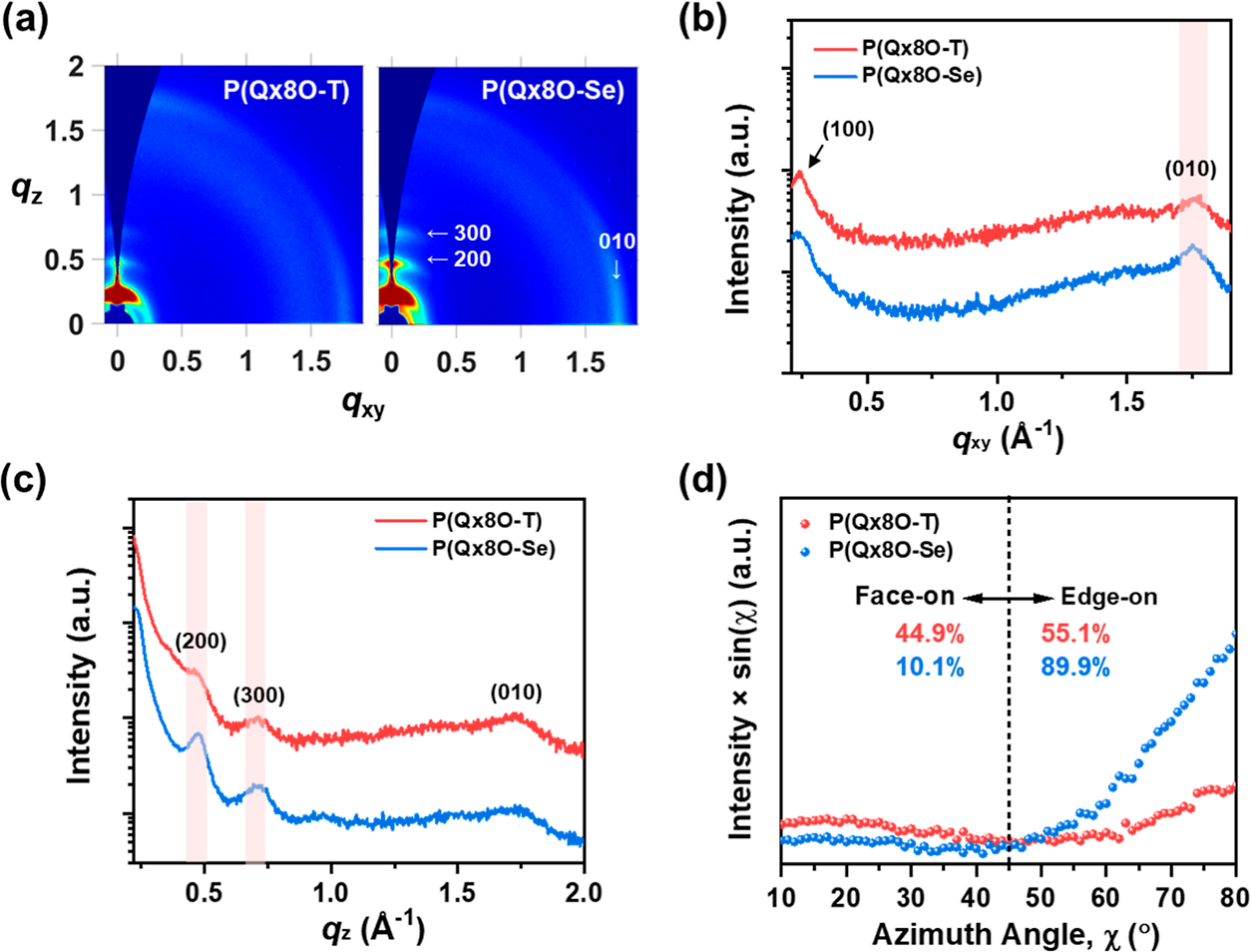
(a) 2D GIWAXS scattering patterns of **P(Qx8O-T)**, **P(Qx8O-Se)**, and **P(NDIDEG-T)** films and their line-cut profiles in the (b) IP and (c) OOP directions. (d) Pole figures were calculated based on the (010) scattering peak intensities of the **P(Qx8O-T)** and **P(Qx8O-Se)** films.

The thin-film crystalline properties of **P(Qx8O-T)** and **P(Qx8O-Se)** were investigated by grazing incidence wide-angle X-ray scattering (GIWAXS) (Figure 2).^53^ All of the polymer films were prepared using water/ethanol mixtures (15:85 v/v) as the processing solvent. As presented in Table 2, **P(Qx8O-T)** and **P(Qx8O-Se)** showed similar (100) lamellar stacking distances (*d*_(100),IP_) and π–π stacking distances (*d*_(010),OOP_) in the in-plane (IP) and out-of-plane (OOP) directions, respectively. However, their polymer packing orientation was different. Although **P(Qx8O-T)** and **P(Qx8O-Se)** both showed mixed face-on and edge-on orientation, the population of the face-on orientation was more dominant in **P(Qx8O-T)** than that in **P(Qx8O-Se)**. The different packing orientation of the polymers is evidenced by pole figures (Figures 2d and S13),^54^ which were calculated based on the (010) scattering peaks (please see the Supporting Information for the detailed calculation). The ratio of the edge-on fraction relative to the face-on fraction (*A*_e_/*A*_f_) was 1.2 for **P(Qx8O-T)**, and 8.9 for **P(Qx8O-Se)** (Table 2), suggesting that **P(Qx8O-T)** has more favorable molecular orientation for vertical charge transport than **P(Qx8O-Se)**.^55^ The relatively planar conformation and stronger aggregation of **P(Qx8O-Se)**, as confirmed by the DFT and TDA results, are considered responsible for its edge-on-dominant behavior, as previously reported by many studies.^20,56^

**Table 2:** Crystalline Properties of **P(Qx8O-T)** and **P(Qx8O-Se)** Thin Films and Their SCLC Hole Mobilities

polymer thin film	*d*_(100),IP_ [Å]^a^	*d*_(010),OOP_ [Å]^a^	*A*_e_/*A*_f_^b^	μ_h_ [cm^2^ V^–1^ s^–1^]^c^
**P(Qx8O-T)**	26.2	3.69	1.2	(3.1 ± 0.5) × 10^–4^
**P(Qx8O-Se)**	26.9	3.72	8.9	(1.6 ± 0.1) × 10^–4^

a Calculated from the GIWAXS line–cut profiles.

b Obtained from pole figures.

c Averaged from five SCLC devices for each system.

The hole transport abilities of the two polymer films were investigated by measurement of the space-charge limited current (SCLC) (Table 2 and Figure S14).^57^ The hole mobilities (μ_h_) of **P(Qx8O-T)** films (μ_h_ = (3.1 ± 0.5) × 10^–4^ cm^2^ V^–1^ s^–1^) were determined to be larger than those of **P(Qx8O-Se)** films (μ_h_ = (1.6 ± 0.1) × 10^–4^ cm^2^ V^–1^ s^–1^). As the structural difference of **P(Qx8O-T)** and **P(Qx8O-Se)** did not greatly affect the π–π stacking distance, the packing orientation of **P(Qx8O-T)** being more advantageous than that of **P(Qx8O-Se)** can explain its higher μ_h_ values.

**Figure 3:**
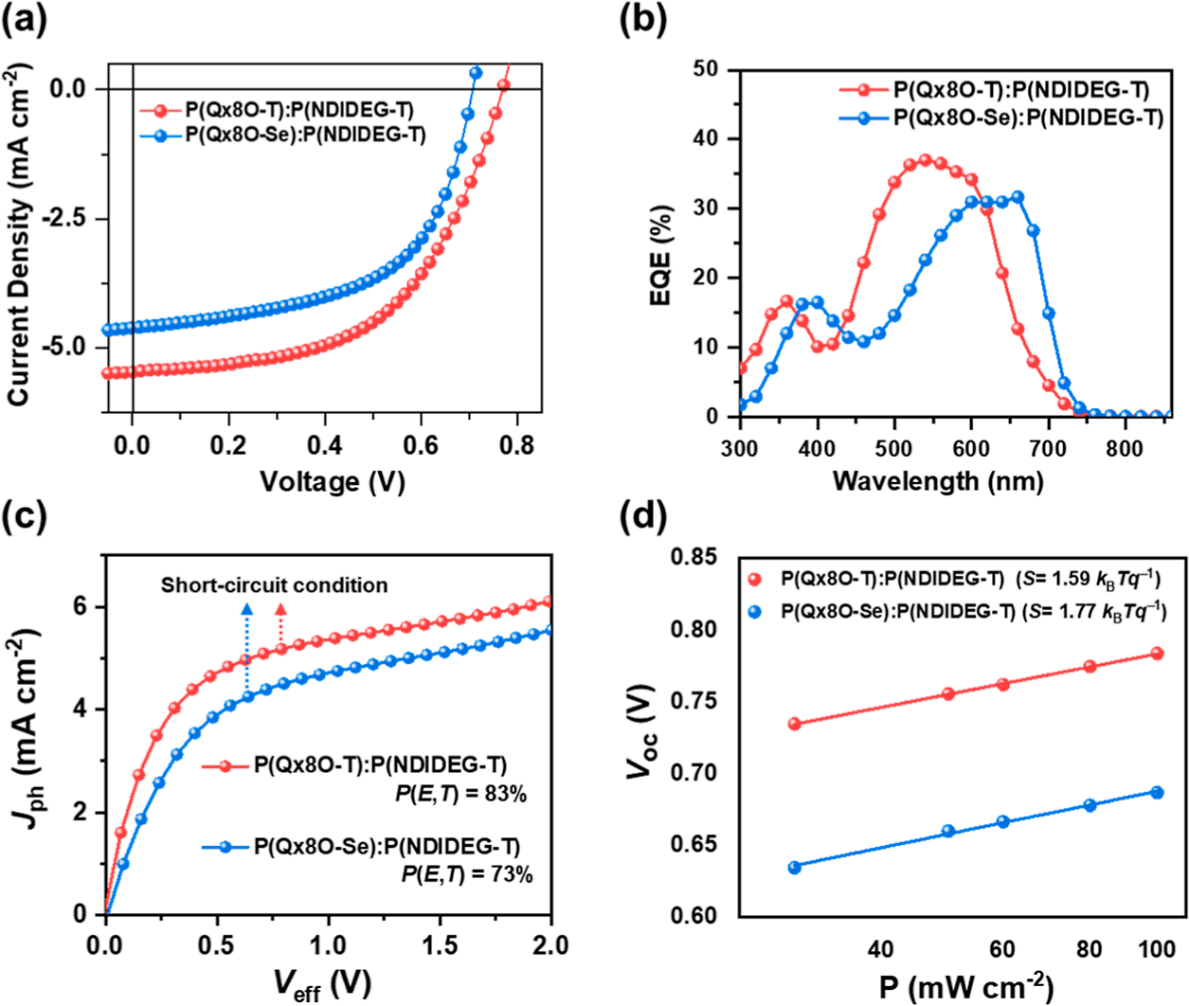
(a) *J*–*V* characteristics under 1 sun illumination, (b) EQE spectra, (c) *J*_ph_ vs *V*_eff_ curves, and (d) *P*-dependent *V*_OC_ of **P(Qx8O-T)**:**P(NDIDEG-T)** and **P(Qx8O-Se)**:**P(NDIDEG-T)** devices.

To investigate the photovoltaic performances of the polymers, aq-APSCs were fabricated with a normal device architecture of indium tin oxide (ITO)/poly(3,4-ethylenedioxythiophene): polystyrenesulfonate (PEDOT:PSS) mixed with 0.15 vol % of 3-glycidoxypropyl-trimethoxysilane (GOPS)/active layer/poly[(9,9′-bis(3′-(*N*,*N*-dimethylamino)-propyl)-2,7-fluorene)-*alt*-5,5′-bis(2,2′-thiophene)-2,6-naphthalene-1,4,5,8-tetracarboxylic-*N*,*N*′-di(2-ethylhexyl)imide] (PNDIT-F3N–Br)/silver (Ag). The optimized donor:acceptor blend ratio was 2:1. Various processing solvents, including acetone, ethyl acetate, and water/ethanol mixtures, were tested (Table S5). Among these solvents, a water/ethanol mixture with a composition of 15:85 (v/v) was the most effective solvent for device fabrication. The active layer of this device has a smooth morphology, while the active layers processed from other water/ethanol mixtures delivered relatively coarser morphologies, as shown in Figure S15. The detailed device fabrication procedure is presented in the Supporting Information. The **P(Qx8O-T)**:**P(NDIDEG-T)**-based aq-APSCs delivered a PCE of 2.27% (*V*_OC_ = 0.78 V, *J*_SC_ = 5.47 mA cm^–2^, and FF = 0.54) (Table 3). Table S6 and Figure S16 compare this performance to previously reported aq-OSCs. Encouragingly, this level of performance is also comparable with the performance of the devices based on PTQ10:N2200 (comparable polymers with alkyl side chains) processed from the halogenated solvent chlorobenzene (best PCE = 2.84%).^58^ The **P(Qx8O-Se)**:**P(NDIDEG-T)**-based devices showed a slightly lower PCE of 1.86% (*V*_OC_ = 0.70 V, *J*_SC_ = 4.69 mA cm^–2^, FF = 0.55) (Figure 3a and Table 2). The different photovoltaic performances mainly originated from the different *V*_OC_ and *J*_SC_ values of the two systems. The higher *V*_OC_ of the **P(Qx8O-T)**-based device is in line with the slightly deeper *E*_HOMO_ of **P(Qx8O-T)** compared to that of **P(Qx8O-Se)**. To the best of our knowledge, the *V*_OC_ reaching nearly 0.8 V is the highest value among aq-APSCs (Table S6 and Figure S16).^18^

**Table 3:** Photovoltaic Properties of aq-APSCs Based on **P(Qx8O-T)**:**P(NDIDEG-T)** and **P(Qx8O-Se)**:**P(NDIDEG-T)** Blends

donor	*V*_OC_ [V]	*J*_SC_ [mA cm^–2^]	calcd *J*_sc_ [mA cm^–2^]	FF	PCE_avg_^a^(PC*E*_max_) [%]
**P(Qx8O-T)**	0.77 ± 0.01	4.82 ± 0.56	5.06	0.55 ± 0.03	2.02 ± 0.16 (2.27)
**P(Qx8O-Se)**	0.70 ± 0.01	4.46 ± 0.23	4.71	0.55 ± 0.02	1.72 ± 0.12 (1.86)

a Calculated from at least 10 devices.

We have also noted that in **P(Qx8O-T)**:**P(NDIDEG-T)**-based devices, different processing solvents yielded different *V*_OC_ values (Table S5). This is a commonly encountered phenomenon when device fabrications involve different processing solvents,^59^ which may be because the changed morphological features by different solvents lead to different charge collection behaviors.^60,61^Figure 3b shows the external quantum efficiency (EQE) spectra of the aq-APSCs. The *J*_SC_s calculated from the EQE curves agree well with the experimental *J*_SC_ values within 6% error.

A series of device characterizations were performed to determine the origin of different *J*_SC_ values of the aq-APSCs. First, the SCLC charge mobilities of the blend films were measured (Table S7). Similar to the hole mobility (μ_h_) trend of pristine films, the **P(Qx8O-T)**-based device showed a higher μ_h_ value [(7.4 ± 1.0) × 10^–5^ cm^2^ V^–1^ s^–1^] than the **P(Qx8O-Se)**-based device [μ_h_ = (3.7 ± 0.8) × 10^–5^ cm^2^ V^–1^ s^–1^]. Analogous to the case of the pristine films, this result can be attributed to the more face-on-oriented polymer structure in the **P(Qx8O-T)**:**P(NDIDEG-T)** blend film (Figure S17), which is favorable for inducing efficient charge transport in the vertical direction. By contrast, in the 2D GIWAXS scattering pattern of the **P(Qx8O-Se)**:**P(NDIDEG-T)** blend film (Figure S17), a pronounced (010) peak in the IP direction together with lamellar scatterings up to (300) peak in the OOP direction was observed, suggesting a stronger edge-on packing contribution than **P(Qx8O-T)**:**P(NDIDEG-T)**. This result partially accounts for the slightly lower PCE of the **P(Qx8O-Se)**-based device than that of the **P(Qx8O-T)**-based device. SCLC characterization of the **P(Qx8O-T)**:**P(NDIDEG-T)** and **P(Qx8O-Se)**:**P(NDIDEG-T)** blends also revealed that the electron mobilities are around 1 order of magnitude lower than the hole mobilities. This high mismatch in mobilities may be the reason for the still lower performance of the devices.^62^

The exciton dissociation probability [*P*(*E*,*T*)] was determined from the relation *P*(*E*,*T*) = *J*_ph,sc_/*J*_ph,sat_, where *J*_ph,sc_ is the photocurrent density at short-circuit condition and *J*_ph,sat_ is the saturated photocurrent density at effective voltage (*V*_eff_ = 2 V) (Figure 3c).^63^ For devices based on **P(Qx8O-T)**:**P(NDIDEG-T)**, *P*(*E*,*T*) (83%) was found to be higher compared to devices based on **P(Qx8O-Se)**:**P(NDIDEG-T)** (*P*(*E*,*T*) = 73%). The higher *P*(*E*,*T*) of **P(Qx8O-T)**:**P(NDIDEG-T)** can be related to its better-mixed blend morphology (*vide infra*) and may also be partially attributed to the larger LUMO–LUMO offset between the donor and acceptor polymers.

The charge recombination properties of the aq-APSCs were also investigated by determining *V*_OC_ and *J*_SC_ values as a function of light intensity (*P*) (Figure 3d and Figure S18).^64^ For **P(Qx8O-T)**:**P(NDIDEG-T)** devices, the lower slope in the *V*_OC_–ln(*P*) plot (*S* = 1.59 *k*_B_*T q*^–1^) and larger slope in the ln(*J*_SC_)–ln(*P*) plot (α = 0.97) than those of the **P(Qx8O-Se)**:**P(NDIDEG-T)** counterparts (*S* = 1.77 *k*_B_*T q*^–1^ and α = 0.94, where *k*_B_ = Boltzmann constant, *T* = absolute temperature, and *q* = elementary charge), suggest that both monomolecular and bimolecular recombination were relatively suppressed. Overall, the higher μ_h_ values suggest more efficient exciton dissociation and better-suppressed recombination of the **P(Qx8O-T)**:**P(NDIDEG-T)** blends than that of the **P(Qx8O-Se)**:**P(NDIDEG-T)** blends, which are consistent with their enhanced *J*_SC_ and PCE.

**Figure 4:**
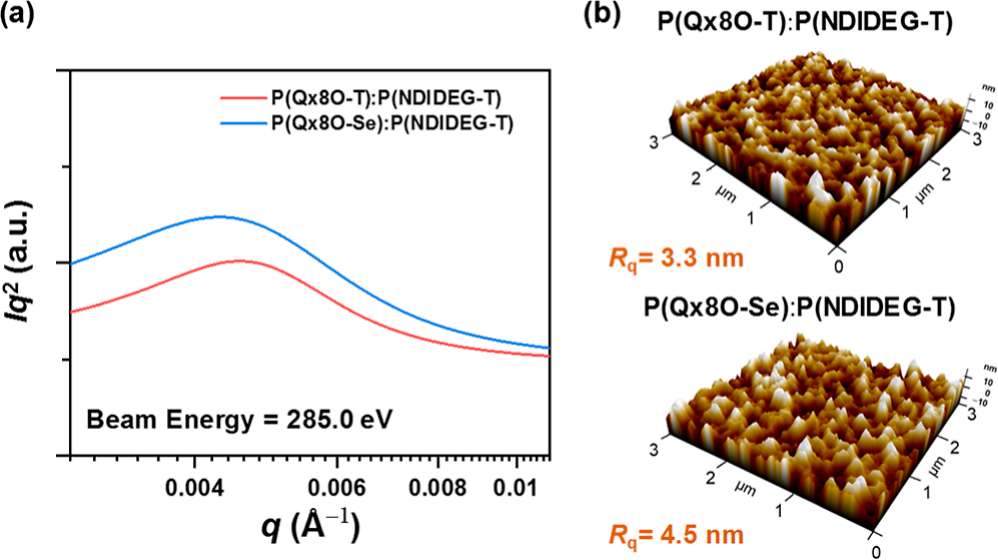
(a) RSoXS profiles of **P(Qx8O-T)**:**P(NDIDEG-T)** and **P(Qx8O-Se)**:**P(NDIDEG-T)** blend films and (b) their AFM height images.

To examine the blend-film morphology of aq-APSCs, resonant soft X-ray scattering (RSoXS) measurements (Figure 4a) were performed.^65^ The beam energy was selected to be 285.0 eV, at which point the scattering contrasts between the donor and acceptor components were maximized. The domain size (*D*_size_) and relative domain purity (*D*_purity_) were calculated from the RSoXS curves (Table S8). The **P(Qx8O-T)**:**P(NDIDEG-T)** blend film showed slightly smaller *D*_size_ (69 nm) and *D*_purity_ (0.92) values than those of **P(Qx8O-Se)**:**P(NDIDEG-T)** (*D*_size_ = 73 nm and *D*_purity_ = 1.00). These data suggest that **P(Qx8O-T)** produced a better-intermixed morphology with smaller domains, when blended with the acceptor polymer, compared to **P(Qx8O-Se)**, which is desirable for efficient charge generation.^66^ In addition, the surfaces of the blend films were probed by AFM (Figure 4b). The AFM height images show that the **P(Qx8O-T)**:**P(NDIDEG-T)** blend formed a smoother film surface than **P(Qx8O-Se)**:**P(NDIDEG-T)**, which may indicate a relatively severe aggregation tendency of the **P(Qx8O-Se)** polymer compared to **P(Qx8O-T)**. Generally, all of the morphological features of the blend films can be associated with the higher *J*_SC_ and photovoltaic performance of the **P(Qx8O-T)**-based system than those of the **P(Qx8O-Se)**-based counterpart.

**Figure 5:**
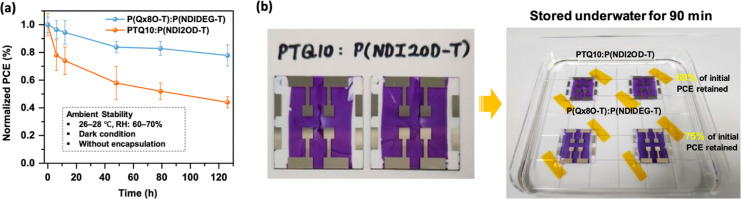
(a) Normalized PCEs of the corresponding devices as a function of time (dark condition, without encapsulation, relative humidity: 60–70%, and temperature: 26–28 °C). (b) Pictures of the OSCs of PTQ10:P(NDI2OD-T) and **P(Qx8O-T)**:**P(NDIDEG-T)** before (left) and after (right) storing the devices underwater for 90 min.

The air-stability of OSCs is crucial for their commercialization. Thus, we tested the stability of the aqueous-processed **P(Qx8O-T)**:**P(NDIDEG-T)** device in dark and ambient conditions without encapsulation. For comparison, a control device fabricated from chloroform using comparable alkylated materials (PTQ10:P(NDI2OD-T) (Figure S10a) was also tested. Consequently, the aqueous-processed **P(Qx8O-T)**:**P(NDIDEG-T)** device showed superior stability by retaining over 80% of the initial performance after 120 h of storage, while the chloroform-processed PTQ10:P(NDI2OD-T device showed less than 50% of the initial PCE in the same period (Figure 5a). Following the arguments previously reported by Kim *et al*.,^18^ the higher air-stability of the aq-OSCs compared to their alkylated counterparts can be ascribed to the strong interactions between the hydrophilic layers, preventing layer delamination over time.

Furthermore, to compare the influence of water on the performance of both devices more intuitively, we monitored their stabilities under underwater conditions. Thus, we observed how the PCEs evolved after immersing both devices in water for 90 min (Figure 5b). Consistently, the aqueous-processed OSC exhibited a slightly better level of stability by retaining 75% of its initial performance after being soaked in water, whereas the PTQ10:P(NDI2OD-T)-based device retained only 69% of the initial PCE value (Table S9). Hence, the stability experiments demonstrate the potential of OEG-grafting to achieve robust devices under ambient and aqueous environments.

## 3 Conclusions

In summary, we have successfully designed and synthesized low-cost and aqueous-processable polymeric donors, namely, **P(Qx8O-T)** and **P(Qx8O-Se)**. These polymers exhibit excellent solubility in eco-friendly water/ethanol mixtures and can be synthesized with minimal synthetic and purification complexity using readily available starting materials. Our DFT simulations suggest that the lower aromatic stabilization energy of the selenophene ring and the large polarizability of the Se-atom are the reasons for the band gap lowering of **P(Qx8O-Se)** compared to its counterpart, **P(Qx8O-T)**. Furthermore, the DFT calculations uncover an intriguing aspect regarding the energy level difference between the OEG-based polymers and their alkylated counterparts that was not previously known. Specifically, it is observed that the OEG side chain has negligible influence on the HOMO/LUMO energy levels of the polymers in a vacuum, with a range of 0.02–0.06 eV. However, when the dielectric environment is taken into account, the OEG side chains exert a significant impact on the energy levels, causing a difference of 0.12–0.17 eV. This finding sheds new light on the role of the OEG side chains in determining the electronic properties of the polymers. When paired with **P(NDIDEG-T)** as the acceptor polymer, the **P(Qx8O-T)**-based aq-APSCs attained a PCE of 2.27%, which is superior to the **P(Qx8O-Se)**-based devices (PCE = 1.86%). The superior performance is mainly ascribed to the favorable packing properties of **P(Qx8O-T)** and its blend morphology, leading to higher μ_h_ values and more efficient charge generation. This photovoltaic performance of **P(Qx8O-T)**:**P(NDIDEG-T)** is the highest among aq-APSCs reported to date, which can be attributed to the design motif of the polymer donors to yield deep HOMO levels (−5.43 eV) and high *V*_OC_ (∼0.8 V). It is also found that the strategy of grafting the corresponding OEG groups over the conjugated backbone enhances the overall stability of the OSC devices, especially in ambient and aqueous environments as compared to the OSCs from their corresponding alkylated counterparts. This study exemplifies the significance of designing inexpensive polymer donors with deep HOMO energy levels, which are processable from eco-friendly solvents, for developing efficient, stable, and commercially viable aq-APSCs. We expect that future development of aqueous-processable acceptors based on state-of-the-art acceptor moieties will possibly fill the PCE gap to realize efficient and eco-friendly OSCs. We believe that our work here will be a valuable input for future studies that aim at the realization of the overall sustainability of the OSCs.

## 4 Experimental Section

### 4.1 Materials/Chemicals

Materials, chemicals, and solvents were obtained from Fisher Chemical, Sigma-Aldrich and other commercial sources and used as received.

### 4.2 Instruments/Characterization

FTIR spectra was generated using the attenuated total reflection-Fourier-transform infrared spectroscopy (ATR-FTIR) technique on a PerkinElmer FT-IR spectrometer. The ^1^H NMR spectra were obtained on a Varian Inova 400 MHz spectrometer at 400.13 and 100.6 MHz, respectively. Mass spectra were obtained on a Xevo G2-XS QTof mass spectrometer equipped with electrospray ionization. Polymer ^13^C NMR spectra were recorded by using a Bruker Avance Neo 600 MHz spectrometer at 150 MHz. A PerkinElmer lambda 1050 UV/vis/NIR spectrometer was used for obtaining the ultraviolet–visible (UV–vis) absorption spectra. A Mettler Toledo TGA/DSC3+ was employed to perform TGA. DSC measurement was performed by using DSC 250 (TA Instruments). Cyclic voltammetry measurements were carried out on a CH Instruments electrochemical workstation from thin films of the polymers on a platinum wire working electrode, Ag/AgCl reference electrode, and another Pt wire as a counter electrode in a solution of tetrabutylammonium hexafluorophosphate in anhydrous acetonitrile. The internal reference was ferrocene/ferrocenium ion (Fc/Fc^+^). The number-average molecular weight (*M*_n_) and dispersity (*D̵*) of the polymers were determined by size-exclusion chromatography (SEC) analyses with an Agilent GPC 1200 instrument equipped with a refractive index detector. The system was calibrated with polystyrene standard, and *ortho*-dichlorobenzene (80 °C) was used as eluent. The AFM images were obtained using a Park Systems model NX10 instrument in the noncontact mode under ambient conditions.

The GIWAXS analysis was conducted at the Pohang Accelerator Laboratory (beamline 9A, Republic of Korea), with incidence angles between 0.12 and 0.14°. The pole figures were acquired using the following procedures:^53,54,67^ first, the GIWAXS line-cut profiles were extracted from every azimuth angle (χ) from 10 to 80° with the interval of 1° (here, χ = 0° indicates the *q*_*z*_ axis). The inaccessible χ ranges near the *q*_*z*_ axis were excluded in this pole figure analysis.^68^ Then, the intensity of the (010) scattering peak in each line–cut profile was multiplied by a geometric factor, sin(χ), and plotted against χ, as shown in Figure S5. Next, scatterings attributable to isotropic domains were subtracted, yielding the final form of the pole figures in Figure 2d. *A*_f_ is defined as the integrated area of the pole figure for 10° < χ < 45°, while *A*_e_ is defined as the integrated area of the pole figure for 45° < χ < 80°, to represent the face-on-oriented and edge-on-oriented fractions, respectively.

The RSoXS experiment was performed at beamline 11.0.1.2 in the Advanced Light Source (United States). Blend films for the measurement were prepared on a 100 nm thick, 1.0 mm × 1.0 mm Si_3_N_4_ membrane supported by a 200 μm thick, 5 mm × 5 mm silicon frame (Norcada Inc.). The domain size (*D*_size_) and relative domain purity (*D*_purity_) of the blend films were calculated from the RSoXS profiles. The *D*_size_ was assumed to be half of the domain spacing (*D*_spacing_), where the *D*_spacing_ is defined as *D*_spacing_ = 2π*q*_peak_^–1^.^69^ Meanwhile, the *D*_purity_ was estimated as the relative value of the square root of the total scattering intensity.^70^

The hole and electron mobilities of the pristine and blend films were measured by the SCLC method using devices with hole-only (ITO/PEDOT:PSS (+0.15 vol % GOPS)/active layer/Au) and electron-only (ITO/zinc oxide (ZnO)/active layer/poly(9,9-bis(3′-(*N*,*N*-dimethyl)-*N*-ethylammoinium-propyl-2,7-fluorene)-*alt*-2,7-(9,9-dioctylfluorene))dibromide (PFN-Br)/Al) device architectures. The thicknesses of the films were ∼80–90 nm. The current–voltage measurements were performed in the voltage range of 0–4 V, and the results were fitted by the Mott–Gurney equation
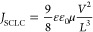
where ε is the relative dielectric constant of the pristine and blend constituents, ε_0_ is the permittivity of free space (8.85 × 10^14^ F cm^–1^), μ is the hole or electron mobility, *L* is the film thickness, and *V* is the potential across the device (*V* = *V*_applied_ – *V*_bi_ – *V*_r_, where *V*_bi_ and *V*_r_ are the voltage drops induced by the built-in potential and the series resistance, respectively).

### 4.3 Polymer Synthesis

The synthesis of the acceptor polymer **P(NDIDEG-T)** was accomplished following a procedure similar to that described in our previous report.^18^ The detailed synthesis of the monomer and intermediates is described in the Supporting Information. The preparations of **P(Qx8O-T)** and **P(Qx8O-Se)** were accomplished by the Stille polymerization reaction between dibromide monomer **7** and 2,5-bis(trimethylstannyl)thiophene and 2,5-bis(trimethylstannyl)selenophene, respectively (Scheme S1).

#### 4.3.1 **P(Qx8O-T)**

Monomer **7** (244.9 mg, 0.34 mmol), 2,5-bis(trimethylstannyl)thiophene (139.3 mg, 0.34 mmol), tris(dibenzylideneacetone)dipalladium(0) (Pd_2_(dba)_3_) (6.2 mg, 0.0068 mmol), and tri(*o*-tolyl)phosphine (P(*o*-Tol)_3_) (8.3 mg, 0.027 mmol) were charged into a 25 mL two-necked round-bottom flask which was degassed and filled with nitrogen five times. Anhydrous toluene (10 mL) was added, and the mixture was heated under reflux for 48 h. The polymer chains were end-capped with 2-(tributylstannyl)thiophene and 2-bromothiophene, respectively. After cooling to room temperature, the crude polymer was precipitated into hexane and filtered into a Soxhlet extraction thimble. Subsequently, the polymer was extracted with hexanes, diethyl ether, methanol, and chloroform. The chloroform fraction was concentrated and precipitated again in hexanes, filtered using a PTFE membrane (0.45 μm), and dried under vacuum at 40 °C overnight to afford **P(Qx8O-T)** (160 mg, 73%).

#### 4.3.2 **P(Qx8O-Se)**

Monomer **7** (193.2 mg, 0.27 mmol), 2,5-bis(trimethylstannyl)selenophene (123.3 mg, 0.27 mmol), Pd_2_(dba)_3_ (4.9 mg, 0.0054 mmol), and P(*o*-Tol)_3_ (6.5 mg, 0.021 mmol) were charged into a microwave vial which was degassed and filled with nitrogen five times. Anhydrous chlorobenzene (1.4 mL) was added, and the reaction mixture was heated at 100 °C for 5 min, 110 °C for 10 min, 140 °C for 50 min, and 150 °C for 1 h in a microwave reactor. The polymer chains were end-capped with 2-(tributylstannyl)thiophene and 2-bromothiophene, respectively. Postpolymerization treatment was similar to that of **P(Qx8O-T)** described above. **P(Qx8O-Se)** (150 mg, 81%) was recovered from the chloroform extract.

## Supporting Information Available

Synthesis, device fabrication, computational detail, and additional data (PDF)

## References

[ref1] Chong K.; Xu X.; Meng H.; Xue J.; Yu L.; Ma W.; Peng Q. Realizing 19.05% Efficiency Polymer Solar Cells by Progressively Improving Charge Extraction and Suppressing Charge Recombination. Adv. Mater. 2022, 34 (13), 2109516 10.1002/adma.202109516. 35080061

[ref2] Gao W.; Qi F.; Peng Z.; Lin F. R.; Jiang K.; Zhong C.; Kaminsky W.; Guan Z.; Lee C.-S.; Marks T. J.; Ade H.; Jen A. K.-Y. Achieving 19% Power Conversion Efficiency in Planar-Mixed Heterojunction Organic Solar Cells Using a Pseudosymmetric Electron Acceptor. Adv. Mater. 2022, 34 (32), 2202089 10.1002/adma.202202089. 35724397

[ref3] Li C.; Zhou J.; Song J.; Xu J.; Zhang H.; Zhang X.; Guo J.; Zhu L.; Wei D.; Han G.; Min J.; Zhang Y.; Xie Z.; Yi Y.; Yan H.; Gao F.; Liu F.; Sun Y. Non-fullerene acceptors with branched side chains and improved molecular packing to exceed 18% efficiency in organic solar cells. Nat. Energy 2021, 6 (6), 605–613. 10.1038/s41560-021-00820-x.

[ref4] Liu Q.; Jiang Y.; Jin K.; Qin J.; Xu J.; Li W.; Xiong J.; Liu J.; Xiao Z.; Sun K.; Yang S.; Zhang X.; Ding L. 18% Efficiency organic solar cells. Sci. Bull. 2020, 65 (4), 272–275. 10.1016/j.scib.2020.01.001. 36659090

[ref5] Wei Y.; Chen Z.; Lu G.; Yu N.; Li C.; Gao J.; Gu X.; Hao X.; Lu G.; Tang Z.; Zhang J.; Wei Z.; Zhang X.; Huang H. Binary Organic Solar Cells Breaking 19% via Manipulating the Vertical Component Distribution. Adv. Mater. 2022, 34 (33), 2204718 10.1002/adma.202204718. 35747988

[ref6] Yuan X.; Zhao Y.; Xie D.; Pan L.; Liu X.; Duan C.; Huang F.; Cao Y. Polythiophenes for organic solar cells with efficiency surpassing 17%. Joule 2022, 6 (3), 647–661. 10.1016/j.joule.2022.02.006.

[ref7] Peng W.; Lin Y.; Jeong S. Y.; Genene Z.; Magomedov A.; Woo H. Y.; Chen C.; Wahyudi W.; Tao Q.; Deng J.; Han Y.; Getautis V.; Zhu W.; Anthopoulos T. D.; Wang E. Over 18% ternary polymer solar cells enabled by a terpolymer as the third component. Nano Energy 2022, 92, 106681 10.1016/j.nanoen.2021.106681.

[ref8] Lin Y.; Zhang Y.; Zhang J.; Marcinskas M.; Malinauskas T.; Magomedov A.; Nugraha M. I.; Kaltsas D.; Naphade D. R.; Harrison G. T.; El-Labban A.; Barlow S.; De Wolf S.; Wang E.; McCulloch I.; Tsetseris L.; Getautis V.; Marder S. R.; Anthopoulos T. D. 18.9% Efficient Organic Solar Cells Based on n-Doped Bulk-Heterojunction and Halogen-Substituted Self-Assembled Monolayers as Hole Extracting Interlayers. Adv. Energy Mater. 2022, 12 (45), 2202503 10.1002/aenm.202202503.

[ref9] Lee S.; Jeong D.; Kim C.; Lee C.; Kang H.; Woo H. Y.; Kim B. J. Eco-Friendly Polymer Solar Cells: Advances in Green-Solvent Processing and Material Design. ACS Nano 2020, 14 (11), 14493–14527. 10.1021/acsnano.0c07488. 33103903

[ref10] Shang L.; Qu S.; Deng Y.; Gao Y.; Yue G.; He S.; Wang Z.; Wang Z.; Tan F. Simple furan-based polymers with the self-healing function enable efficient eco-friendly organic solar cells with high stability. J. Mater. Chem. C 2022, 10 (2), 506–516. 10.1039/D1TC05111C.

[ref11] Sun R.; Wang T.; Luo Z.; Hu Z.; Huang F.; Yang C.; Min J. Achieving Eco-Compatible Organic Solar Cells with Efficiency > 16.5% Based on an Iridium Complex-Incorporated Polymer Donor. Sol. RRL 2020, 4 (7), 2000156 10.1002/solr.202000156.

[ref12] Chen H.; Zhang R.; Chen X.; Zeng G.; Kobera L.; Abbrent S.; Zhang B.; Chen W.; Xu G.; Oh J.; Kang S.-H.; Chen S.; Yang C.; Brus J.; Hou J.; Gao F.; Li Y.; Li Y. A guest-assisted molecular-organization approach for > 17% efficiency organic solar cells using environmentally friendly solvents. Nat. Energy 2021, 6 (11), 1045–1053. 10.1038/s41560-021-00923-5.

[ref13] McDowell C.; Bazan G. C. Organic solar cells processed from green solvents. Curr. Opin. Green Sustainable Chem. 2017, 5, 49–54. 10.1016/j.cogsc.2017.03.007.

[ref14] Ma Z.; Zhao B.; Gong Y.; Deng J.; Tan Z. a. Green-solvent-processable strategies for achieving large-scale manufacture of organic photovoltaics. J. Mater. Chem. A 2019, 7 (40), 22826–22847. 10.1039/C9TA09277C.

[ref15] United States Environmental Protection Agency. U. S. Environmental Protection Agency, Toxics Release Inventory (TRI) Program. http://www.epa.gov/toxics-release-inventory-tri-program/tri-listed-chemicals (accessed February 2023).

[ref16] Pan X.; Sharma A.; Kroon R.; Gedefaw D.; Elmas S.; Yin Y.; Andersson G. G.; Lewis D. A.; Andersson M. R. Water/Ethanol Soluble p-Type Conjugated Polymers for the Use in Organic Photovoltaics. Front. Mater. Sci. 2020, 7, 281 10.3389/fmats.2020.00281.

[ref17] Shang L.; Zhang W.; Zhang B.; Gao Y.; He S.; Dong G.; Li W.; Bai H.; Yue G.; Chen S.; Tan F. Ethanol-Processable Polyfuran Derivative for Eco-Friendly Fabrication of Organic Solar Cells Featuring Self-Healing Function. Sol. RRL 2022, 6 (10), 2200605 10.1002/solr.202200605.

[ref18] Lee S.; Kim Y.; Wu Z.; Lee C.; Oh S. J.; Luan N. T.; Lee J.; Jeong D.; Zhang K.; Huang F.; Kim T.-S.; Woo H. Y.; Kim B. J. Aqueous-Soluble Naphthalene Diimide-Based Polymer Acceptors for Efficient and Air-Stable All-Polymer Solar Cells. ACS Appl. Mater. Interfaces 2019, 11 (48), 45038–45047. 10.1021/acsami.9b13812. 31701742

[ref19] Lee C.; Lee H. R.; Choi J.; Kim Y.; Nguyen T. L.; Lee W.; Gautam B.; Liu X.; Zhang K.; Huang F.; Oh J. H.; Woo H. Y.; Kim B. J. Efficient and Air-Stable Aqueous-Processed Organic Solar Cells and Transistors: Impact of Water Addition on Processability and Thin-Film Morphologies of Electroactive Materials. Adv. Energy Mater. 2018, 8 (34), 1802674 10.1002/aenm.201802674.

[ref20] Jeong D.; Jo I.-Y.; Lee S.; Kim J. H.; Kim Y.; Kim D.; Reynolds J. R.; Yoon M.-H.; Kim B. J. High-Performance n-Type Organic Electrochemical Transistors Enabled by Aqueous Solution Processing of Amphiphilicity-Driven Polymer Assembly. Adv. Funct. Mater. 2022, 32 (16), 2111950 10.1002/adfm.202111950.

[ref21] Nguyen T. L.; Lee C.; Kim H.; Kim Y.; Lee W.; Oh J. H.; Kim B. J.; Woo H. Y. Ethanol-Processable, Highly Crystalline Conjugated Polymers for Eco-Friendly Fabrication of Organic Transistors and Solar Cells. Macromolecules 2017, 50 (11), 4415–4424. 10.1021/acs.macromol.7b00452.

[ref22] Kim Y.; Choi J.; Lee C.; Kim Y.; Kim C.; Nguyen T. L.; Gautam B.; Gundogdu K.; Woo H. Y.; Kim B. J. Aqueous Soluble Fullerene Acceptors for Efficient Eco-Friendly Polymer Solar Cells Processed from Benign Ethanol/Water Mixtures. Chem. Mater. 2018, 30 (16), 5663–5672. 10.1021/acs.chemmater.8b02086.

[ref23] Kim C.; Kang H.; Choi N.; Lee S.; Kim Y.; Kim J.; Wu Z.; Woo H. Y.; Kim B. J. C70-based aqueous-soluble fullerene for the water composition-tolerant performance of eco-friendly polymer solar cells. J. Mater. Chem. C 2020, 8 (43), 15224–15233. 10.1039/D0TC03049J.

[ref24] Yu Y.; Dong C.; Alahmadi A. F.; Meng B.; Liu J.; Jäkle F.; Wang L. A p-π* conjugated triarylborane as an alcohol-processable n-type semiconductor for organic optoelectronic devices. J. Mater. Chem. C 2019, 7 (24), 7427–7432. 10.1039/C9TC01562K.

[ref25] Kukhta N. A.; Marks A.; Luscombe C. K. Molecular Design Strategies toward Improvement of Charge Injection and Ionic Conduction in Organic Mixed Ionic-Electronic Conductors for Organic Electrochemical Transistors. Chem. Rev. 2022, 122 (4), 4325–4355. 10.1021/acs.chemrev.1c00266. 34902244 PMC8874907

[ref26] Wang E.; Hou L.; Wang Z.; Hellström S.; Zhang F.; Inganäs O.; Andersson M. R. An Easily Synthesized Blue Polymer for High-Performance Polymer Solar Cells. Adv. Mater. 2010, 22 (46), 5240–5244. 10.1002/adma.201002225. 20827685

[ref27] Wu Y.; Zheng Y.; Yang H.; Sun C.; Dong Y.; Cui C.; Yan H.; Li Y. Rationally pairing photoactive materials for high-performance polymer solar cells with efficiency of 16.53%. Sci. China: Chem. 2020, 63 (2), 265–271. 10.1007/s11426-019-9599-1.

[ref28] Han D.; Han Y.; Kim Y.; Lee J.-W.; Jeong D.; Park H.; Kim G.-U.; Kim F. S.; Kim B. J. Efficient, thermally stable poly(3-hexylthiophene)-based organic solar cells achieved by non-covalently fused-ring small molecule acceptors. J. Mater. Chem. A 2022, 10 (2), 640–650. 10.1039/D1TA09392D.

[ref29] Nguyen T. L.; Choi H.; Ko S. J.; Uddin M. A.; Walker B.; Yum S.; Jeong J. E.; Yun M. H.; Shin T. J.; Hwang S.; Kim J. Y.; Woo H. Y. Semi-crystalline photovoltaic polymers with efficiency exceeding 9% in a ∼ 300 nm thick conventional single-cell device. Energy Environ. Sci. 2014, 7 (9), 3040–3051. 10.1039/C4EE01529K.

[ref30] Liao S.-H.; Jhuo H.-J.; Cheng Y.-S.; Chen S.-A. Fullerene Derivative-Doped Zinc Oxide Nanofilm as the Cathode of Inverted Polymer Solar Cells with Low-Bandgap Polymer (PTB7-Th) for High Performance. Adv. Mater. 2013, 25 (34), 4766–4771. 10.1002/adma.201301476. 23939927

[ref31] Zhao W.; Qian D.; Zhang S.; Li S.; Inganäs O.; Gao F.; Hou J. Fullerene-Free Polymer Solar Cells with over 11% Efficiency and Excellent Thermal Stability. Adv. Mater. 2016, 28 (23), 4734–4739. 10.1002/adma.201600281. 27061511

[ref32] Rech J. J.; Neu J.; Qin Y.; Samson S.; Shanahan J.; Josey R. F.; Ade H.; You W. Designing Simple Conjugated Polymers for Scalable and Efficient Organic Solar Cells. ChemSusChem 2021, 14 (17), 3561–3568. 10.1002/cssc.202100910. 34008311

[ref33] Neu J.; Samson S.; Ding K.; Rech J. J.; Ade H.; You W. Oligo(ethylene glycol) Side Chain Architecture Enables Alcohol-Processable Conjugated Polymers for Organic Solar Cells. Macromolecules 2023, 56 (5), 2092–2103. 10.1021/acs.macromol.2c02259.

[ref34] Genene Z.; Lee J.-W.; Lee S.-W.; Chen Q.; Tan Z.; Abdulahi B. A.; Yu D.; Kim T.-S.; Kim B. J.; Wang E. Polymer Acceptors with Flexible Spacers Afford Efficient and Mechanically Robust All-Polymer Solar Cells. Adv. Mater. 2022, 34 (6), 2107361 10.1002/adma.202107361. 34820914

[ref35] Genene Z.; Mammo W.; Wang E.; Andersson M. R. Recent Advances in n-Type Polymers for All-Polymer Solar Cells. Adv. Mater. 2019, 31 (22), 1807275 10.1002/adma.201807275. 30790384

[ref36] Ding X.; Chen X.; Xu Y.; Ni Z.; He T.; Qiu H.; Li C.-Z.; Zhang Q. A selenophene-containing near-infrared unfused acceptor for efficient organic solar cells. Chem. Eng. J. 2022, 429, 132298 10.1016/j.cej.2021.132298.

[ref37] Zhuang W.; Zhen H.; Kroon R.; Tang Z.; Hellström S.; Hou L.; Wang E.; Gedefaw D.; Inganäs O.; Zhang F.; Andersson M. R. Molecular orbital energy level modulation through incorporation of selenium and fluorine into conjugated polymers for organic photovoltaic cells. J. Mater. Chem. A 2013, 1 (43), 13422–13425. 10.1039/c3ta13040a.

[ref38] Kim K.-H.; Park S.; Yu H.; Kang H.; Song I.; Oh J. H.; Kim B. J. Determining Optimal Crystallinity of Diketopyrrolopyrrole-Based Terpolymers for Highly Efficient Polymer Solar Cells and Transistors. Chem. Mater. 2014, 26 (24), 6963–6970. 10.1021/cm502991d.

[ref39] Filate T. T.; Tang S.; Genene Z.; Edman L.; Mammo W.; Wang E. Hydrophilic Conjugated Polymers for Sustainable Fabrication of Deep-Red Light-Emitting Electrochemical Cells. Adv. Mater. Technol. 2024, 9 (3), 2301696 10.1002/admt.202301696.

[ref40] Fan B.; Lin F.; Wu X.; Zhu Z.; Jen A. K. Y. Selenium-Containing Organic Photovoltaic Materials. Acc. Chem. Res. 2021, 54 (20), 3906–3916. 10.1021/acs.accounts.1c00443. 34606230

[ref41] Lu T.; Chen F. Quantitative analysis of molecular surface based on improved Marching Tetrahedra algorithm. J. Mol. Graphics Modell. 2012, 38, 314–323. 10.1016/j.jmgm.2012.07.004. 23085170

[ref42] Sanderson R. T. Principles of electronegativity Part I. General nature. J. Chem. Educ. 1988, 65 (2), 112 10.1021/ed065p112.

[ref43] Little E. J. Jr.; Jones M. M. A complete table of electronegativities. J. Chem. Educ. 1960, 37 (5), 231 10.1021/ed037p231.

[ref44] Chen X.; Zhang Z.; Ding Z.; Liu J.; Wang L. Diketopyrrolopyrrole-based Conjugated Polymers Bearing Branched Oligo(Ethylene Glycol) Side Chains for Photovoltaic Devices. Angew. Chem., Int. Ed. 2016, 55 (35), 10376–10380. 10.1002/anie.201602775. 27258171

[ref45] Marenich A. V.; Cramer C. J.; Truhlar D. G. Universal Solvation Model Based on Solute Electron Density and on a Continuum Model of the Solvent Defined by the Bulk Dielectric Constant and Atomic Surface Tensions. J. Phys. Chem. B 2009, 113 (18), 6378–6396. 10.1021/jp810292n. 19366259

[ref46] Rousseva S.; Besten H. d.; van Kooij F. S.; Doting E. L.; Doumon N. Y.; Douvogianni E.; Anton Koster L. J.; Hummelen J. C. Reaching a Double-Digit Dielectric Constant with Fullerene Derivatives. J. Phys. Chem. C 2020, 124 (16), 8633–8638. 10.1021/acs.jpcc.0c01390.

[ref47] Wang X.-Y.; Liu Y.; Wang Z.-Y.; Lu Y.; Yao Z.-F.; Ding Y.-F.; Yu Z.-D.; Wang J.-Y.; Pei J. Revealing the effect of oligo(ethylene glycol) side chains on n-doping process in FBDPPV-based polymers. J. Polym. Sci. 2022, 60 (3), 538–547. 10.1002/pol.20210493.

[ref48] Sworakowski J.; Janus K. On the reliability of determination of energies of HOMO levels in organic semiconducting polymers from electrochemical measurements. Org. Electron. 2017, 48, 46–52. 10.1016/j.orgel.2017.05.031.

[ref49] Chen H.-Y.; Yeh S.-C.; Chen C.-T.; Chen C.-T. Comparison of thiophene- and selenophene-bridged donor-acceptor low band-gap copolymers used in bulk-heterojunction organic photovoltaics. J. Mater. Chem. 2012, 22 (40), 21549–21559. 10.1039/c2jm33735e.

[ref50] Fan B.; Lin F.; Oh J.; Fu H.; Gao W.; Fan Q.; Zhu Z.; Li W. J.; Li N.; Ying L.; Huang F.; Yang C.; Jen A. K.-Y. Enabling High Efficiency of Hydrocarbon-Solvent Processed Organic Solar Cells through Balanced Charge Generation and Non-Radiative Loss. Adv. Energy Mater. 2021, 11 (41), 2101768 10.1002/aenm.202101768.

[ref51] Li D.; Guo C.; Zhang X.; Du B.; Yu C.; Wang P.; Cheng S.; Wang L.; Cai J.; Wang H.; Liu D.; Yao H.; Sun Y.; Hou J.; Wang T. Non-fullerene acceptor pre-aggregates enable high efficiency pseudo-bulk heterojunction organic solar cells. Sci. China: Chem. 2022, 65 (2), 373–381. 10.1007/s11426-021-1128-1.

[ref52] Carlotti B.; Cai Z.; Kim H.; Sharapov V.; Madu I. K.; Zhao D.; Chen W.; Zimmerman P. M.; Yu L.; Goodson T. Charge Transfer and Aggregation Effects on the Performance of Planar vs Twisted Nonfullerene Acceptor Isomers for Organic Solar Cells. Chem. Mater. 2018, 30 (13), 4263–4276. 10.1021/acs.chemmater.8b01047.

[ref53] Rivnay J.; Mannsfeld S. C. B.; Miller C. E.; Salleo A.; Toney M. F. Quantitative Determination of Organic Semiconductor Microstructure from the Molecular to Device Scale. Chem. Rev. 2012, 112 (10), 5488–5519. 10.1021/cr3001109. 22877516

[ref54] Kim Y.; Park H.; Abdilla A.; Yun H.; Han J.; Stein G. E.; Hawker C. J.; Kim B. J. Chain-Length-Dependent Self-Assembly Behaviors of Discrete Conjugated Oligo(3-hexylthiophene). Chem. Mater. 2020, 32 (8), 3597–3607. 10.1021/acs.chemmater.0c00869.

[ref55] Aubry T. J.; Ferreira A. S.; Yee P. Y.; Aguirre J. C.; Hawks S. A.; Fontana M. T.; Schwartz B. J.; Tolbert S. H. Processing Methods for Obtaining a Face-On Crystalline Domain Orientation in Conjugated Polymer-Based Photovoltaics. J. Phys. Chem. C 2018, 122 (27), 15078–15089. 10.1021/acs.jpcc.8b02859.

[ref56] Jeon G. G.; Lee M.; Nam J.; Park W.; Yang M.; Choi J.-H.; Yoon D. K.; Lee E.; Kim B.; Kim J. H. Simple Solvent Engineering for High-Mobility and Thermally Robust Conjugated Polymer Nanowire Field-Effect Transistors. ACS Appl. Mater. Interfaces 2018, 10 (35), 29824–29830. 10.1021/acsami.8b07643. 30088908

[ref57] Chiguvare Z.; Dyakonov V. Trap-limited hole mobility in semiconducting poly(3-hexylthiophene). Phys. Rev. B 2004, 70 (23), 235207 10.1103/PhysRevB.70.235207.

[ref58] Zhou K.; Xian K.; Qi Q.; Gao M.; Peng Z.; Liu J.; Liu Y.; Li S.; Zhang Y.; Geng Y.; Ye L. Unraveling the Correlations between Mechanical Properties, Miscibility, and Film Microstructure in All-Polymer Photovoltaic Cells. Adv. Funct. Mater. 2022, 32 (30), 2201781 10.1002/adfm.202201781.

[ref59] Chen Z.; Yan L.; Rech J. J.; Hu J.; Zhang Q.; You W. Green-Solvent-Processed Conjugated Polymers for Organic Solar Cells: The Impact of Oligoethylene Glycol Side Chains. ACS Appl. Polym. Mater. 2019, 1 (4), 804–814. 10.1021/acsapm.9b00044.

[ref60] Naveed H. B.; Zhou K.; Ma W. Interfacial and Bulk Nanostructures Control Loss of Charges in Organic Solar Cells. Acc. Chem. Res. 2019, 52 (10), 2904–2915. 10.1021/acs.accounts.9b00331. 31577121

[ref61] Poelking C.; Benduhn J.; Spoltore D.; Schwarze M.; Roland S.; Piersimoni F.; Neher D.; Leo K.; Vandewal K.; Andrienko D. Open-circuit voltage of organic solar cells: interfacial roughness makes the difference. Commun. Phys. 2022, 5 (1), 307 10.1038/s42005-022-01084-x.

[ref62] Cao B.; He X.; Sorge J. B.; Lalany A.; Ahadi K.; Afshar A.; Olsen B. C.; Hauger T. C.; Mobarok M. H.; Li P.; Cadien K. C.; Brett M. J.; Luber E. J.; Buriak J. M. Understanding the Effects of a High Surface Area Nanostructured Indium Tin Oxide Electrode on Organic Solar Cell Performance. ACS Appl. Mater. Interfaces 2017, 9 (44), 38706–38715. 10.1021/acsami.7b10610. 29022714

[ref63] Blom P. W. M.; Mihailetchi V. D.; Koster L. J. A.; Markov D. E. Device Physics of Polymer:Fullerene Bulk Heterojunction Solar Cells. Adv. Mater. 2007, 19 (12), 1551–1566. 10.1002/adma.200601093.

[ref64] Cowan S. R.; Roy A.; Heeger A. J. Recombination in polymer-fullerene bulk heterojunction solar cells. Phys. Rev. B 2010, 82 (24), 245207 10.1103/PhysRevB.82.245207.

[ref65] Sun C.; Lee J.-W.; Lee C.; Lee D.; Cho S.; Kwon S.-K.; Kim B. J.; Kim Y.-H. Dimerized small-molecule acceptors enable efficient and stable organic solar cells. Joule 2023, 7 (2), 416–430. 10.1016/j.joule.2023.01.009.

[ref66] Jiao X.; Ye L.; Ade H. Quantitative Morphology-Performance Correlations in Organic Solar Cells: Insights from Soft X-Ray Scattering. Adv. Energy Mater. 2017, 7 (18), 1700084 10.1002/aenm.201700084.

[ref67] Vaselabadi S. A.; Shakarisaz D.; Ruchhoeft P.; Strzalka J.; Stein G. E. Radiation damage in polymer films from grazing-incidence X-ray scattering measurements. J. Polym. Sci., Part B: Polym. Phys. 2016, 54 (11), 1074–1086. 10.1002/polb.24006.

[ref68] Hammond M. R.; Kline R. J.; Herzing A. A.; Richter L. J.; Germack D. S.; Ro H.-W.; Soles C. L.; Fischer D. A.; Xu T.; Yu L.; Toney M. F.; DeLongchamp D. M. Molecular Order in High-Efficiency Polymer/Fullerene Bulk Heterojunction Solar Cells. ACS Nano 2011, 5 (10), 8248–8257. 10.1021/nn202951e. 21939254

[ref69] Mu C.; Liu P.; Ma W.; Jiang K.; Zhao J.; Zhang K.; Chen Z.; Wei Z.; Yi Y.; Wang J.; Yang S.; Huang F.; Facchetti A.; Ade H.; Yan H. High-Efficiency All-Polymer Solar Cells Based on a Pair of Crystalline Low-Bandgap Polymers. Adv. Mater. 2014, 26 (42), 7224–7230. 10.1002/adma.201402473. 25238661

[ref70] Gu X.; Zhou Y.; Gu K.; Kurosawa T.; Guo Y.; Li Y.; Lin H.; Schroeder B. C.; Yan H.; Molina-Lopez F.; Tassone C. J.; Wang C.; Mannsfeld S. C. B.; Yan H.; Zhao D.; Toney M. F.; Bao Z. Roll-to-Roll Printed Large-Area All-Polymer Solar Cells with 5% Efficiency Based on a Low Crystallinity Conjugated Polymer Blend. Adv. Energy Mater. 2017, 7 (14), 1602742 10.1002/aenm.201602742.

