# Supplementary material for: Aqueous Processed All-Polymer Solar Cells with High Open-Circuit Voltage Based on Low-Cost Thiophene–Quinoxaline Polymers
Source: ACS Appl Mater Interfaces. 2024 Mar 1;16(10):12886–96. doi: 10.1021/acsami.3c18994 (PMC10941072; doi:10.1021/acsami.3c18994)
Supplement: Supplementary file 1 — am3c18994_si_001.pdf [file am3c18994_si_001.pdf]

Supporting Information

Aqueous Processed All-Polymer Solar Cells with High Open-Circuit Voltage Based on Low-Cost Thiophene-Quinoxaline Polymers

Tadele T. Filate,[a,b,∇] Seungjin Lee,[c,d,∇] Leandro R. Franco,[e] Qiaonan Chen,[a] Zewdneh Genene,[a] Cleber F.N. Marchiori,[e] Yoonjoo Lee,[f] Moyses Araujo,[e,g] Wendimagegn Mammo,[b,*] Han Young Woo,[f,*] Bumjoon J. Kim,[c,*] and Ergang Wang[a,*]

[a]Department of Chemistry and Chemical Engineering, Chalmers University of Technology, SE-412 96 Göteborg, Sweden.

[b]Department of Chemistry, Addis Ababa University, P.O. Box 33658, Addis Ababa, Ethiopia.

[c]Department of Chemical and Biomolecular Engineering, Korea Advanced Institute of Science and Technology (KAIST), Daejeon 34141, Republic of Korea.

[d]Energy Materials Research Center, Korea Research Institute of Chemical Technology (KRICT), Daejeon 34114, Republic of Korea.

[e]Department of Engineering and Physics, Karlstad University, 65188 Karlstad, Sweden.

[f]Department of Chemistry, Korea University, Seoul 02841, Republic of Korea.

[g]Materials Theory Division, Department of Physics and Astronomy, Uppsala University, 75120 Uppsala, Sweden.

[*]Email: wendimagegn.mammo@aau.edu.et (W.M.)

[*]Email: hywoo@korea.ac.kr (H.Y.W.)

[*]Email: bumjoonkim@kaist.ac.kr (B.J.K.)

[*]Email: ergang@chalmers.se (E.W.)

Table of Contents

Synthesis of Monomer and Intermediates

Computational Details

Fabrication of aq-APSC Devices

Supporting Scheme and Figures

Scheme S1. Synthetic routes for the preparation of compound **3**, monomer **7**, and polymers **P(Qx8O-T)** and **P(Qx8O-Se)**.

Figure S1. ^{1}H NMR spectra of **P(Qx8O-T)** and **P(Qx8O-Se)**.

Figure S2. ^{13}C NMR spectra of **P(Qx8O-T)** and **P(Qx8O-Se)**.

Figure S3. (a) TGA thermograms, (b) DSC second heating/cooling cycles of **P(Qx8O-T)** and **P(Qx8O-Se)**.

Figure S4. Normalized cyclic voltammograms of **P(Qx8O-T)** and **P(Qx8O-Se)**.

Figure S5: Kohn Sham frontier molecular orbitals of the trimeric donor and acceptor polymers. Orbitals plotted with an isodensity value of 0.04 electrons/$Å^3$.

Figure S6. Electrostatic surface potential (ESP) maps of the trimeric donor and acceptor materials. EPS were calculated using the Multiwfn[1, 2] program.

Figure S7. ESP surfaces for thiophene (left) and selenophene (right).

Figure S8. (a) Normalized cyclic voltammogram and (b) normalized UV-vis absorption spectrum of **PTQ10**.

Figure S9. Ground State relaxed geometries (at HSE03/6-31G(d,p) theory level) of the trimeric donor and acceptor polymers.

Figure S10. (a) Chemical structures of donor and acceptor polymers. (b) Energy levels of the donors and acceptor polymers, with the OEG and alkyl side chains, obtained from CV experiment and the Gibbs free energies of the oxidation and reduction reactions at the HSE03/6-311G(d,p) theory level. The HOMO and LUMO values of P(NDI2OD-T) was taken from the reported literature.[3]

Figure S11. TD-DFT electronic excitations (orange vertical lines) of the donors and acceptor polymers in the UV-vis range. Experimental spectra plotted for comparison. Electron/hole picture of the first electronic transition (S_1) plotted with an isodensity value of 0.04 electrons/$Å^3$.

Figure S12. Temperature-dependent UV–vis absorption spectra of (a) **P(Qx8O-T)** and (b) **P(Qx8O-Se)** in water/ethanol (15:85 v/v) mixtures (0.02 mg mL^{-1}).

Figure S13. Pole figures obtained at (010) scattering peaks of (a) **P(Qx8O-T**) and (b) **P(Qx8O-Se**) films. The peak intensities were multiplied by a geometric factor, sin(χ).

Figure S14. SCLC curves of pristine (a) **P(Qx8O-T)** and (b) **P(Qx8O-Se)** films.

Figure S15. AFM height images of the **P(Qx8O-T)**:**P(NDIDEG-T)** blend films depending on the H_2O:EtOH volume ratio in EtOH95 (H_2O:EtOH, 5:95 (v/v)), EtOH85 (H_2O:EtOH, 15:85 (v/v)), ETOH75 (H_2O:EtOH, 25:75 (*v/v*)), and EtOH65 (H_2O:EtOH, 35:65 (v/v)) (scale bars: 1 μm).
Figure S16. A plot of V_{OC} *vs* PCE of aq-OSCs (left) and performance of aq-OSCs reported to date.
Figure S17. (a) 2D GIWAXS scattering patterns of **P(Qx8O-T)**:**P(NDIDEG-T)** and **P(Qx8O-Se)**:**P(NDIDEG-T)** blend films and **P(NDIDEG-T)** pristine film. Their line-cut profiles in the (b) IP and (c) OOP directions.
Figure S18. *P*-dependent J_{sc} of **P(Qx8O-T)**:**P(NDIDEG-T)** and **P(Qx8O-Se)**:**P(NDIDEG-T)** devices.

Notes

Note 1: **Selenophene and thiophene under perspective**

Note 2. **Conformational preferences of thiophene and selenophene rings relative to acceptor units**

Supporting Tables

Table S1. Cost of materials for the synthesis of 1 g of **P(Qx8O-T)**.

Table S2. Cost of materials for the synthesis of 1 g of **P(Qx8O-Se)**.

Table S3 Summary of the electronic properties of the trimeric donor and acceptor polymers at the HSE03/6-311G(d,p) theory level.

Table S4. Optical and electrochemical properties of **PTQ10**.

Table S5. Photovoltaic performance of **P(Qx8O-T)**:**P(NDIDEG-T)** (donor:acceptor = 2:1 w/w) in various processing solvents.

Table S6. Summary of OSC performances of devices processed from water/ethanol (15:85 v/v).

Table S7. SCLC hole or electron mobilities of blend films and **P(NDIDEG-T)** pristine film.

Table S8. Domain size and relative domain purity values of **P(Qx8O-T)**:**P(NDIDEG-T)** and **P(Qx8O-Se)**:**P(NDIDEG-T)** calculated from the RSoXS profiles.
Table S9. Photovoltaic performance of PTQ10:P(NDI2OD-T)- and **P(Qx8O-T)**:**P(NDIDEG-T)**-based devices before and after immersing the devices underwater.

Synthesis of Monomer and Intermediates

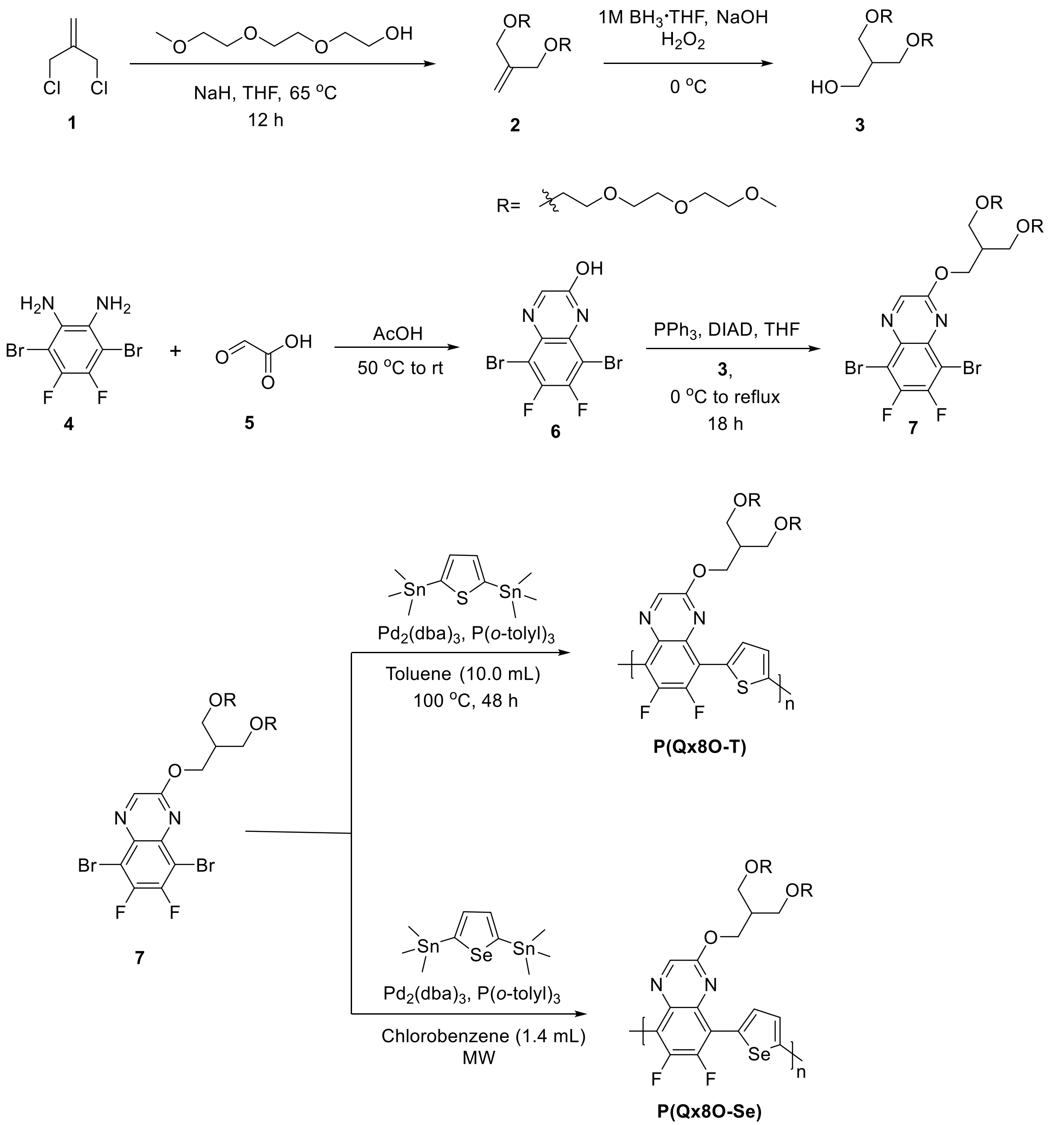

Scheme S1. Synthetic routes for the preparation of compound **3**, monomer **7**, and polymers **P(Qx8O-T)** and **P(Qx8O-Se)**.

13-Methylene-2,5,8,11,15,18,21,24-octaoxapentacosane (2)

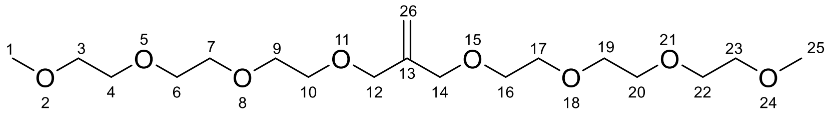

2

The synthesis of compound **2** was conducted following a literature procedure.[4-6] Thus, 3-chloro-2-(chloromethyl)prop-1-ene (10.0 g, 80 mmol) and sodium hydride (60%, 10.0 g, 250 mmol) were mixed in THF (180 mL). Triethylene glycol monomethyl ether (38.4 mL, 39.4 g, 0.24 mol) was then added dropwise at room temperature. The resulting mixture was heated under reflux overnight. After cooling to room temperature, the reaction was quenched with distilled water and extracted with dichloromethane (DCM). The organic extract was washed with brine three times and was then dried over anhydrous sodium sulfate and concentrated by rotary evaporation. Gradient column chromatography over silica gel was conducted to purify the crude reaction product (petroleum ether to acetone:DCM (1:1)). Compound **2** was obtained as colorless oil (29.5 g, 97%). FT-IR ν_{max} = 2862, 1656, 1451, 1095 cm^{-1}. ^{1}H NMR (400 MHz, $CDCl_3$) δ 5.15 (2H, *s*, H26), 3.98 (4H, s, H12, H14), 3.66 – 3.58 (16H, *m*, H6, H7, H9, H10, H16, H17, H19, H20), 3.58 – 3.49 (8H, *m*, H3, H4, H22, H23), 3.34 (6H, *s*, H1, H25). ^{13}C NMR (101 MHz, $CDCl_3$) δ 142.4 (C13), 114.1 (C26), 71.9 (C12, C14), 71.8 (C3, C23), 70.6 (C6, C7, C19, C20), 70.5 (C10, C16), 70.5 (C4, C22),, 69.4 (C9, C17), 59.0 (C1, C25).

13-(2,5,8,11-tetraoxadodecyl)-2,5,8,11-tetraoxatetradecan-14-ol (3)

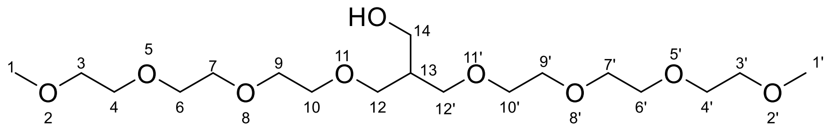

3

Compound **2** (28.25 g, 74.25 mmol) was dissolved in THF (180 mL). The solution was cooled to 0 ^{o}C and BH_3 (1 M in THF, 76 mL) was added dropwise over 40 min. The reaction was warmed to room temperature and stirred for 3 h and was quenched with 3 M NaOH at 0 ^{o}C. Hydrogen peroxide (30%, 57 mL) was added and the mixture was stirred at room temperature for

1 h. The reaction was saturated with potassium carbonate and extracted with DCM. The organic layer was dried and passed through a short silica pad to afford compound **3** as colorless oil (28.2 g, 95.2%). HRMS (ESI+, APCI+) calcd for $C_{18}H_{38}O_9$ $[M+H]^+$ 399.2594, found: 399.2601. FT-IR ν_{max} = 3486, 2871, 1092 cm^{-1}. ^{1}H NMR (400 MHz, $CDCl_3$) δ 3.69 (2H, *t*, *J* = 5.3 Hz, H14), 3.62 – 3.49 (28H, *m*, H3, H4, H6, H7, H9, H10, H12, H3', H4', H6', H7', H9', H10', H12'), 3.34 (6H, *s*, H1, H1'), 3.02 (1H, *t*, *J* = 6.0 Hz -OH), 2.16 – 1.98 (1H, *m*, H13). ^{13}C NMR (101 MHz, $CDCl_3$) δ 71.9 (C3, C3'), 70.8 (C12, C12'), 70.6 (C10, C10'), 70.5 (C6, C7, C6', C7'), 70.5 (C4, C4'), 70.4, (C9, C9'), 63.4 (C14), 59.0 (C1, C1'), 41.2 (C13).

5,8-Dibromo-6,7-difluoroquinoxalin-2-ol (6)

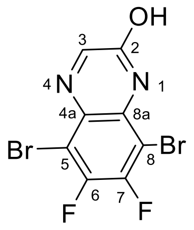

6

Compound **5** (1.71 g, 5.66 mmol) was dissolved in acetic acid (56.6 mL) and glyoxylic acid (0.62 mL, 50 wt% in H_2O) was added dropwise. The reaction mixture was warmed up to 45 ^oC and stirred for 10 min. It was then cooled to room temperature and stirred overnight. The white precipitate (1.9 g) was collected by filtration and the product was used in the next reaction without further purification.[7]

2-((13-(2,5,8,11-tetraoxadodecyl)-2,5,8,11-tetraoxatetradecan-14-yl)oxy)-5,8-dibromo-6,7-difluoroquinoxaline (7)

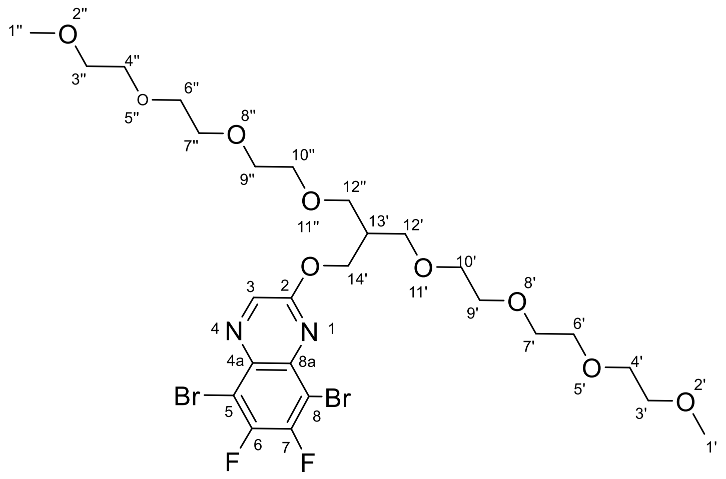

7

Compound **6** (0.30 g, 0.88 mmol) and PPh_3 (0.35 g, 1.33 mmol) were added into a 100 mL two-necked round bottom flask. The mixture was degassed and purged with nitrogen three times. THF (26 mL) was then added and the mixture was cooled to 0 °C. Compound **3** (0.50 g, 1.26 mmol) was added followed by dropwise addition of diisopropyl azodicarboxylate (0.26 mL, 0.27 g, 1.34 mmol). The light-yellow solution was warmed up to room temperature and then refluxed for 18 h. The reaction mixture was cooled to room temperature and was quenched with distilled water and extracted with DCM. The combined DCM extract was washed with distilled water and brine. The organic phase was dried over anhydrous magnesium sulfate and the solvent was removed by rotary evaporation.[8] The crude product was recrystallized from petroleum ether/ethyl acetate mixture to remove the triphenylphosphine oxide. Silica gel column chromatography using ethyl acetate as eluent afforded compound **7** (0.4 g, 63% over two steps). HRMS (ESI+, APCI+) calcd for $C_{26}H_{38}Br_2F_2N_2O_9$ $[M+H]^+$ 719.099, found: 719.0999. FT-IR ν_{max} = 2871, 1706, 1402, 1101, 636 cm^{-1}. ^{1}H NMR (400 MHz, $CDCl_3$) δ 8.50 (1H, *s*, H3), 4.63 (2H, *d*, *J* = 5.7 Hz, H14'), 3.77 – 3.59 (24H, *m*, H3', H3", H4', H4", H6', H6", H7', H7", H9', H9", H10', H10"), 3.61 – 3.43 (4H, *m*, H12', H12"), 3.37 (6H, *s*, H1', H1"), 2.68 – 2.29 (1H, *m*, H13'). ^{13}C NMR (101 MHz, $CDCl_3$) δ 158.4 (C2), 151.9 (C7), 149.6 (C6), 147.0 (C3), 140.5 (C8a), 136.2 (C4a), 109.8 (C5), 107.7 (C8), 71.8 (C3', C3"), 70.6 (C9, C9'), 70.5 (C4', C4"), 70.5 (C6', C6"), 70.4 (C10', C10", C12', C12"), 69.2 (C7', C7"), 66.1 (C14'), 59.0 (C1', C1"), 39.2 (C13').

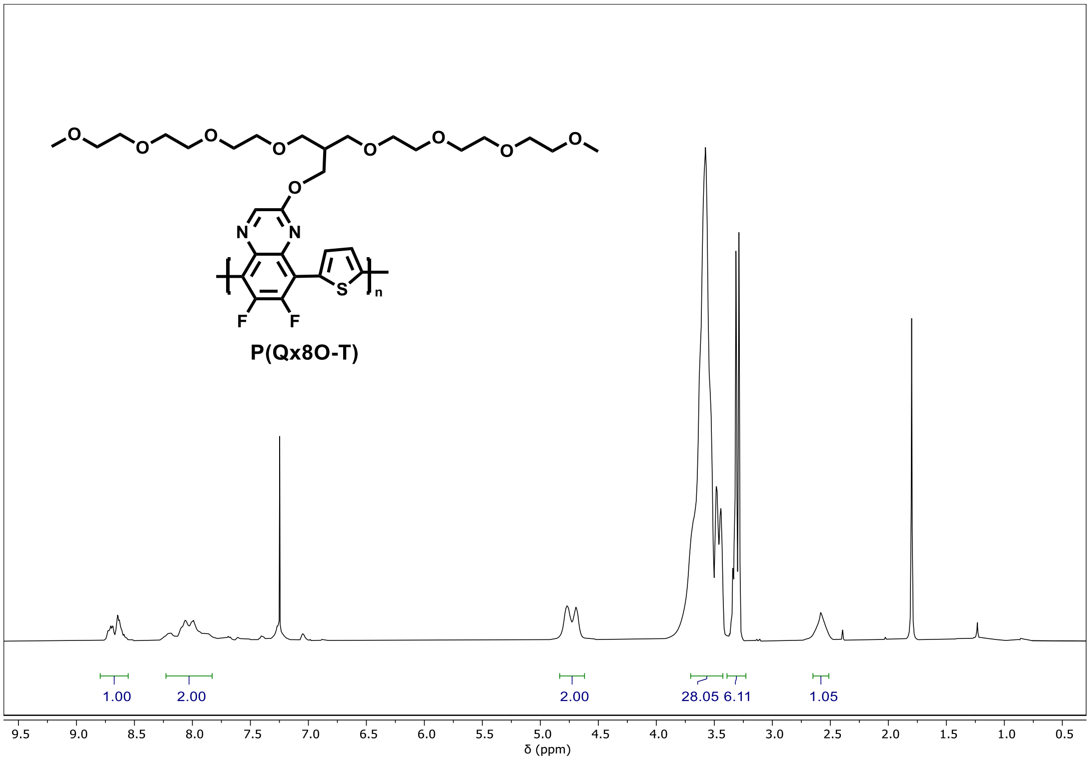

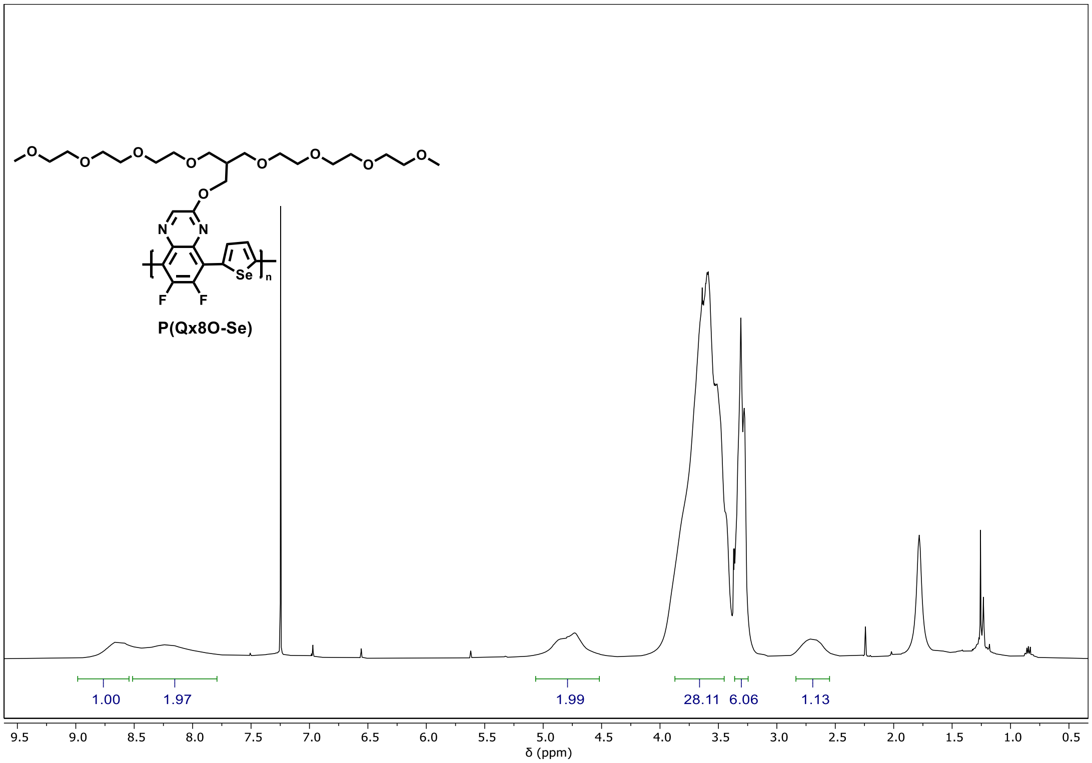

Figure S1. ^{1}H NMR spectra of **P(Qx8O-T)** and **P(Qx8O-Se)**.

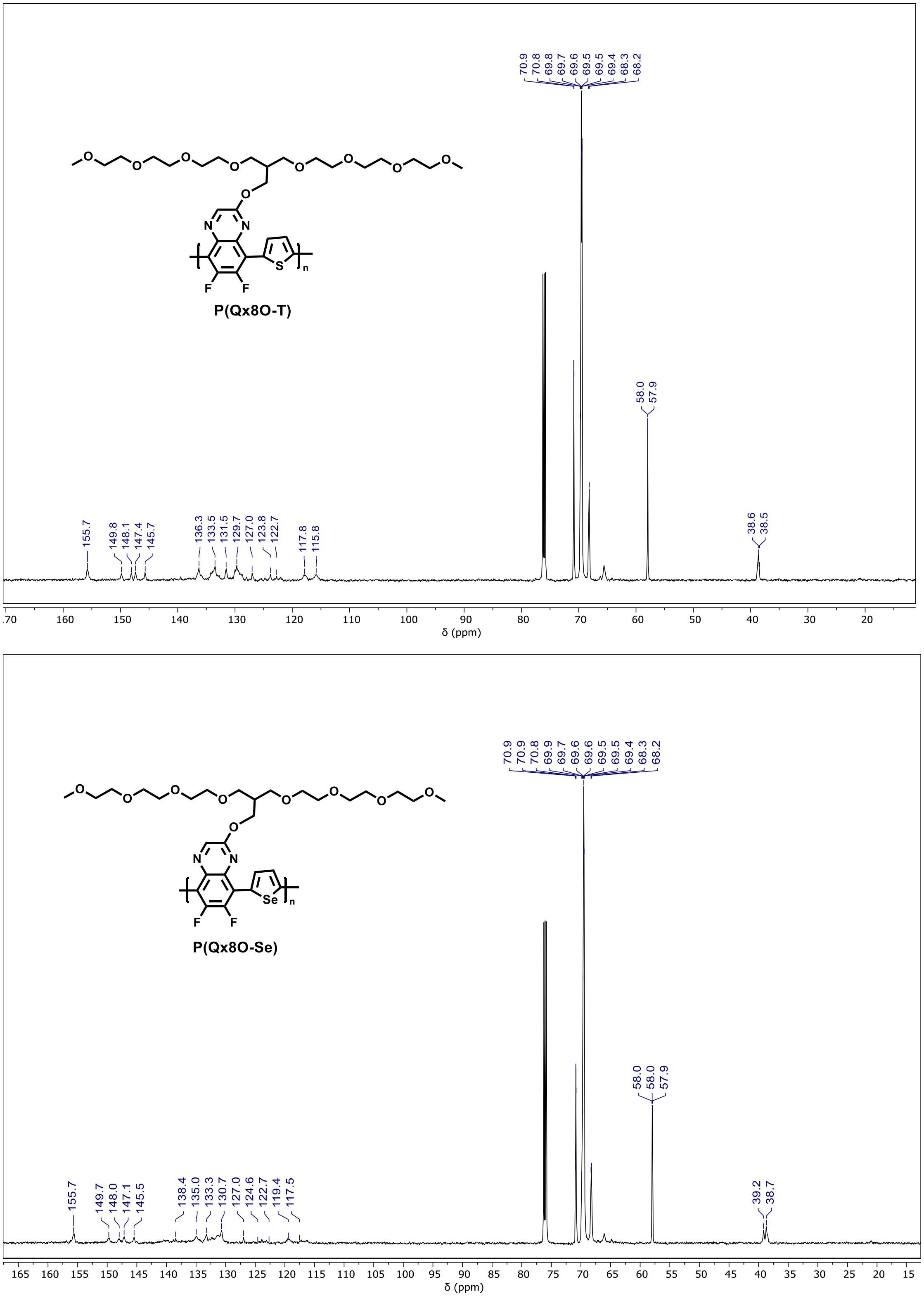

Figure S2. ^{13}C NMR spectra of **P(Qx8O-T)** and **P(Qx8O-Se)**.

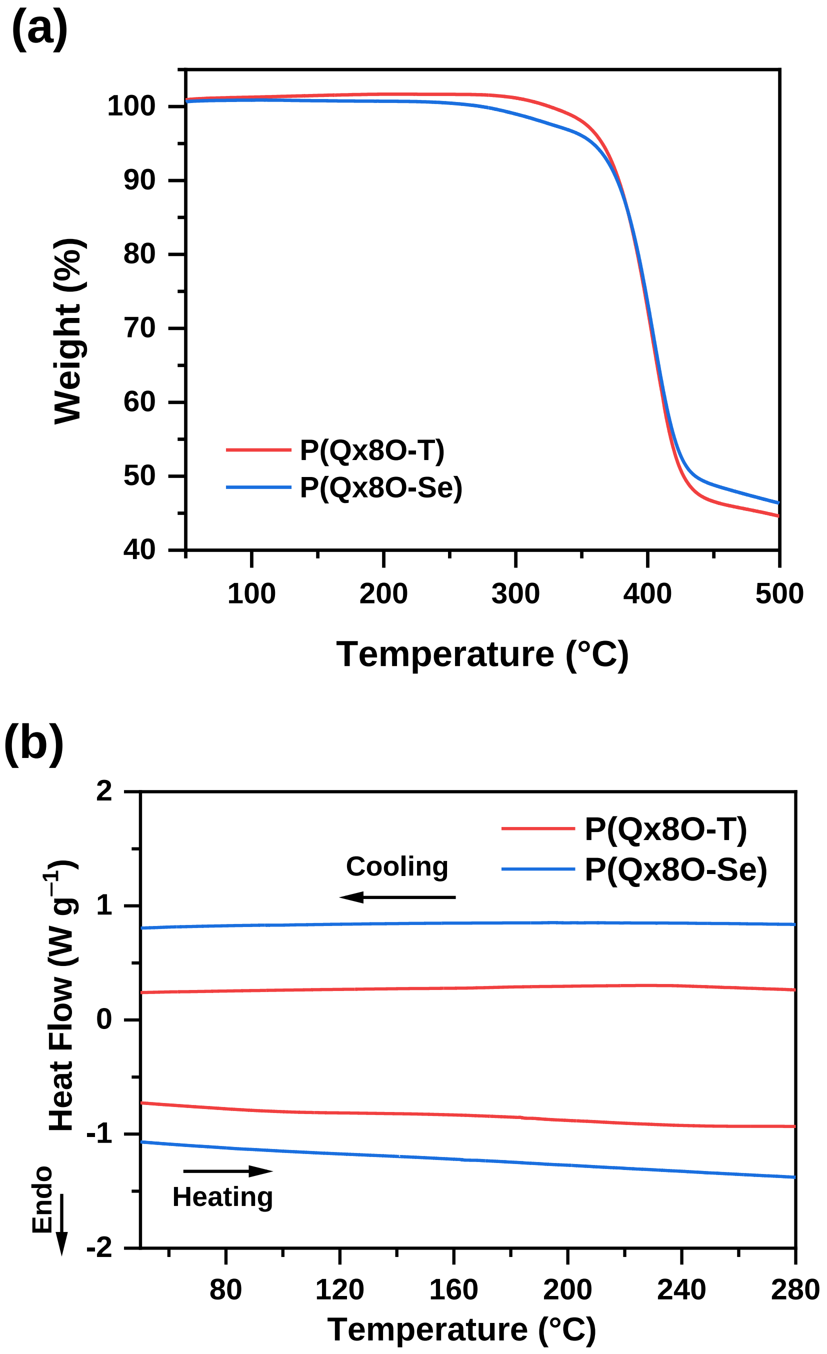

Figure S3. (a) TGA thermograms, (b) DSC second heating/cooling cycles of **P(Qx8O-T)** and **P(Qx8O-Se)**.

Table S1. Cost of materials for the synthesis of 1 g of **P(Qx8O-T)**.

**Name of chemical**	**Amount (mmol)**	**Cost ($)**
1,4-Dibromo-2,3-difluorobenzene	5.56	6.93
(Fuming) Nitric acid	5.56	0.08
Trifluoromethanesulfonic acid (Triflic acid)	189.20	9.89
Acetic acid	0.08[a)]	1.61
Iron (powder)	66.22	0.35
Sodium hydroxide	295.63	0.27
Glyoxylic acid	4.68	0.30
Tetrahydrofuran	0.13[a)]	1.55
Diisopropyl azodicarboxylate (DIAD)	4.46	0.81
Triphenylphosphine	3.28	0.08
Thiophene	3.41	0.03
n-Butyllithium (2.5M)	6.99	0.27
Trimethyltin chloride (1M)	7.15	10.78
Toluene	0.03	0.08
P(***o***-tol)$_3$	0.43	1.88
$Pd_2dba_3.CHCl_3$	0.05	5.54
Borane tetrahydrofuran complex solution (1 M)	4.61[b)]	3.81
Hydrogen peroxide (30%)	4.00[b)]	0.15
Potassium carbonate	14.50	0.05
3-Chloro-2-(chloromethyl)prop-1-ene	5.68	0.08
Sodium hydride (60%)	17.60	0.14
Triethylene glycol monomethyl ether	17.00	0.24
**Total cost for P(Qx8O-T)**		**44.89**

[a)]volume (L) [b)] volume (mL)

Table S2. Cost of materials for the synthesis of 1 g of **P(Qx8O-Se)**.

**Name of chemical**	**Amount (mmol)**	**Cost ($)**
1,4-Dibromo-2,3-difluorobenzene	5.56	6.93
(Fuming) Nitric Acid	5.56	0.08
Trifluoromethanesulfonic acid (Triflic acid)	189.20	9.89
Acetic acid	0.08[a)]	1.61
Iron (powder)	66.22	0.35
Sodium hydroxide	295.63	0.27
Glyoxylic acid	4.68	0.30
Tetrahydrofuran	0.13[a)]	1.55
Diisopropyl azodicarboxylate (DIAD)	4.46	0.81
Triphenylphosphine	3.28	0.08
Selenophene	3.41	10.22
n-Butyllithium (2.5M)	6.99	0.27
Trimethyltin chloride (1M)	7.15	10.78
Toluene	0.03	0.08
P(***o***-tol)$_3$	0.43	1.88
$Pd_2dba_3.CHCl_3$	0.05	5.54
Borane tetrahydrofuran complex solution (1 M)	4.61[b)]	3.81
Hydrogen peroxide (30%)	4.00[b)]	0.15
Potassium carbonate	14.50	0.05
3-Chloro-2-(chloromethyl)prop-1-ene	5.68	0.08
Sodium hydride (60%)	17.60	0.14
Triethylene glycol monomethyl ether	17.00	0.24
**Total cost for P(Qx8O-Se)**		**55.08**

[a)]volume (L) [b)] volume (mL)

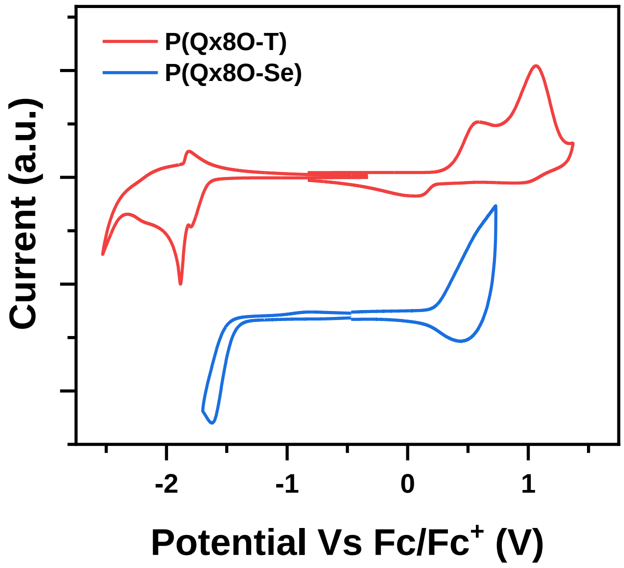

Figure S4. Normalized cyclic voltammograms of **P(Qx8O-T)** and **P(Qx8O-Se)**.

Computational Details

The electronic structures of the two donor polymers, **P(Qx8O-T)** and **P(Qx8O-Se)**, and the acceptor polymer, **P(NDIDEG-T)**, were investigated using Density Functional Theory (DFT) and its time-dependent variant (TD-DFT), combining the HSE03 (Heyd-Scuseria-Ernzerhof)[9] exchange-correlation (XC) functional and the basis sets 6-31G(d,p) (only for geometry relaxation and Gibbs free energy corrections) and 6-311G(d,p).[10] The polymers were represented as single chains comprising three monomers. In capturing the essential properties of polymers, this approach strikes a balance between computational efficiency and accuracy. To account for the side chain effects, the donors were modelled with both the OEG and alkyl groups, by simply replacing the glycol oxygens with CH_2. To ensure accurate characterization of the electronic properties of the polymers, their geometries were fully relaxed in the ground state (GS). To account for the impact of the surrounding environment, the implicit universal solvation model SMD was employed.[11] We have used two solvents, n-octanol (ε = 9.86) and 1-fluorooctane (ε = 3.89), in order to mimic as closely as possible the dielectric constants of the polymeric films. Specifically, n-octanol was utilized for OEG-based polymers while 1-fluorooctane was selected for alkyl-based polymers.

These choices were made based on previous research studies.[12-14] Moreover, the full Gibbs free energies of the oxidation and reduction reactions have been calculated to compare with the experimentally estimated HOMO and LUMO energies, respectively. All the calculations were performed using the Gaussian 16 program (Rev C.01).[15]

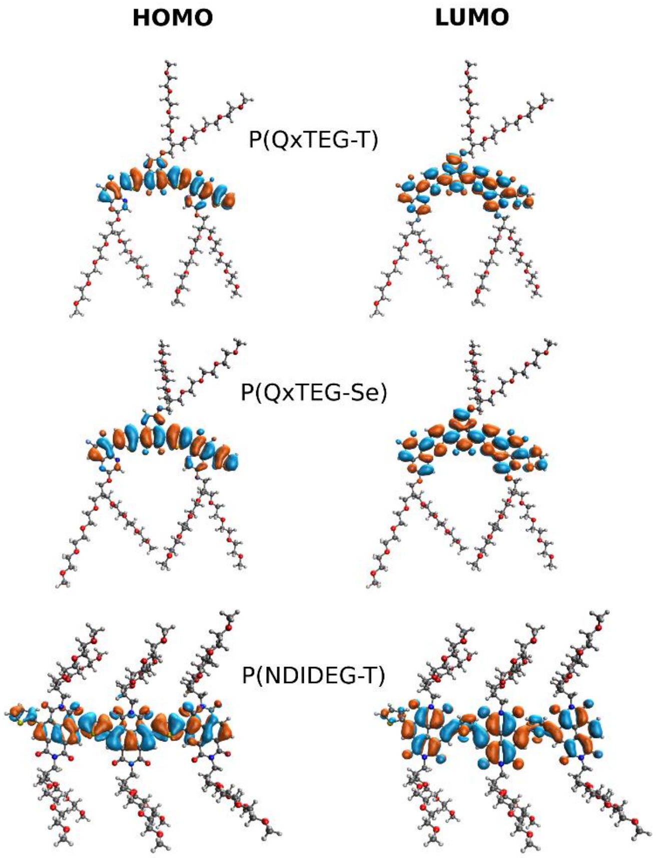

Figure S5: Kohn Sham frontier molecular orbitals of the trimeric donor and acceptor polymers. Orbitals plotted with an isodensity value of 0.04 electrons/Å^3.

Table S3 Summary of the electronic properties of the trimeric donor and acceptor polymers at the HSE03/6-311G(d,p) theory level.

Properties	P(Qx8O-T)	P(QxC27-T)	P(Qx8O-Se)	P(QxC27-Se)	P(NDIDEG-T)	P(NDIC40-T)
E_{LUMO} (eV)	−3.28	−3.16	−3.37	−3.23	−4.26	−4.02
E_{HOMO} (eV)	−5.50	−5.67	−5.41	−5.57	−6.08	−6.20
Bandgap (eV)	2.22	2.51	2.04	2.34	1.82	2.18
Dipole (Debye) [a)]	4.90	3.39	5.50	4.26	16.00	3.36
Isotropic Polarizability (a.u.)	1927.9	2109.5	2060.9	2220.1	2765.6	2959.6
Hirshfeld average charge on S or Se atom	0.04	0.04	0.13	0.13	0.05	0.05
MPI	13.35	6.71	13.82	6.78	14.84	7.32
λ_{max} absorption (nm)	560.9	576.3	604.9	618.7	627.9	620.9

[a)] Dipole moment is a very sensitive property to the charge distribution of the system, and it is molecular conformation-dependent, thus the side chain positioning in the relaxed geometry is mainly responsible for the large dipole moment of P(NDIDEG-T). Environment effects were considered for all calculated properties, employing the SMD model, as described above.

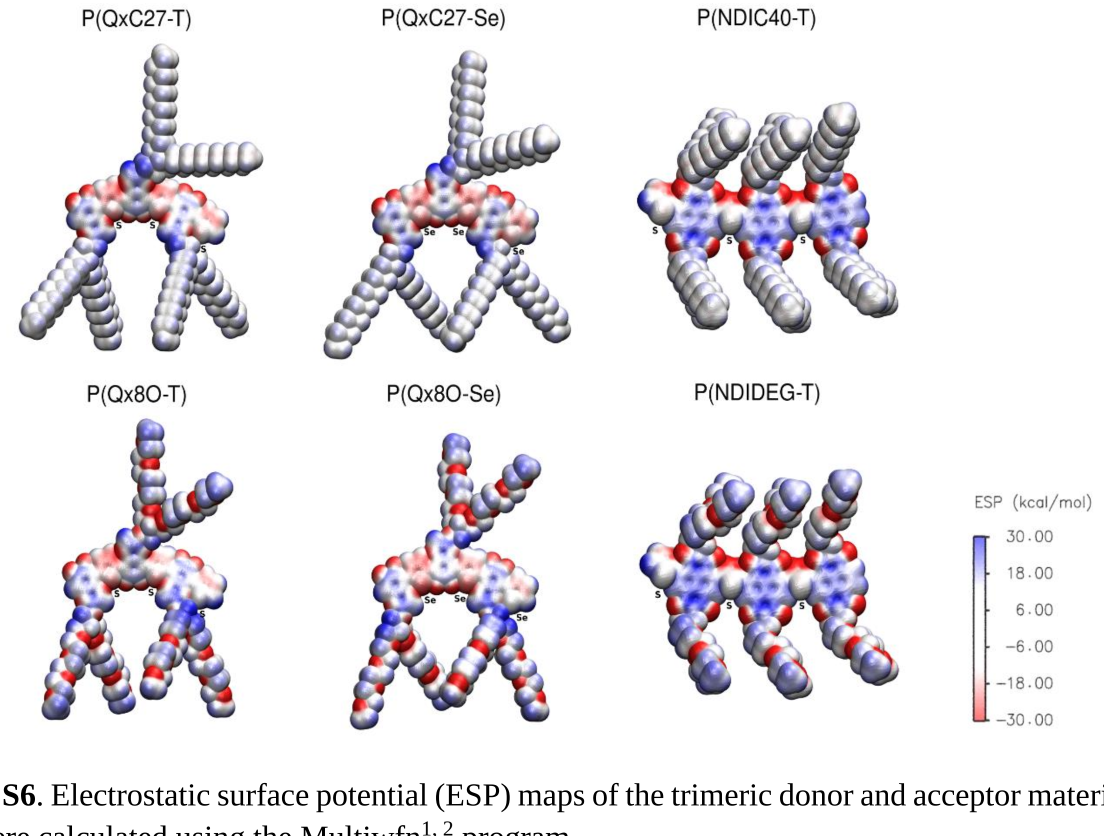

Figure S6. Electrostatic surface potential (ESP) maps of the trimeric donor and acceptor materials. EPS were calculated using the Multiwfn[1, 2] program.

Note 1: **Selenophene and thiophene under perspective**

In order to explore the electronegativity of the S and Se atoms, we carried out a short study in simpler systems: the thiophene (Dipole = 0.55 Db, Atomic Hirshfeld charge on S atom: 0.04) and the selenophene (Dipole = 0.34 Db, Atomic Hirshfeld charge on Se atom: 0.13) rings. We can see that the dipole moment is higher for thiophene than selenophene, while the atomic charge on S atom is less positive than in Se atom, following the trend of the electronegativity of these atoms. However, ESP is more negative (slightly more red) near the Se atom (**Figure S17**), compared to S atom. The MPI for selenophene (8.9 kcal mol^{-1}) is also greater than thiophene (8.7 kcal mol^{-1}). The ESP depends on the distribution of electrons within the molecule, as well as the nuclear charges of the atoms. Se is a larger atom than S, with a larger atomic radius (around 103 pm for S, and 119 pm for Se) and more diffused electron cloud. This leads to a more negative electrostatic potential near Se compared to S.

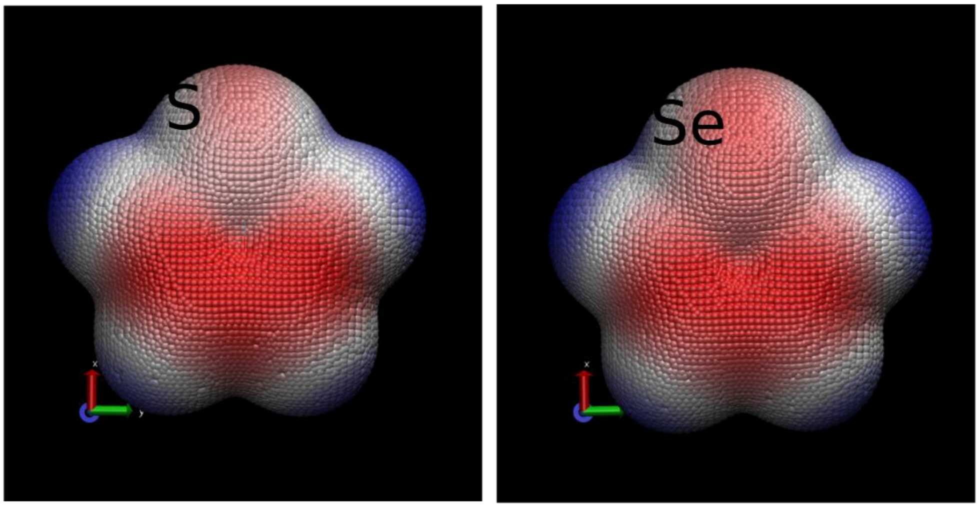

Figure S7. ESP surfaces for thiophene (left) and selenophene (right).

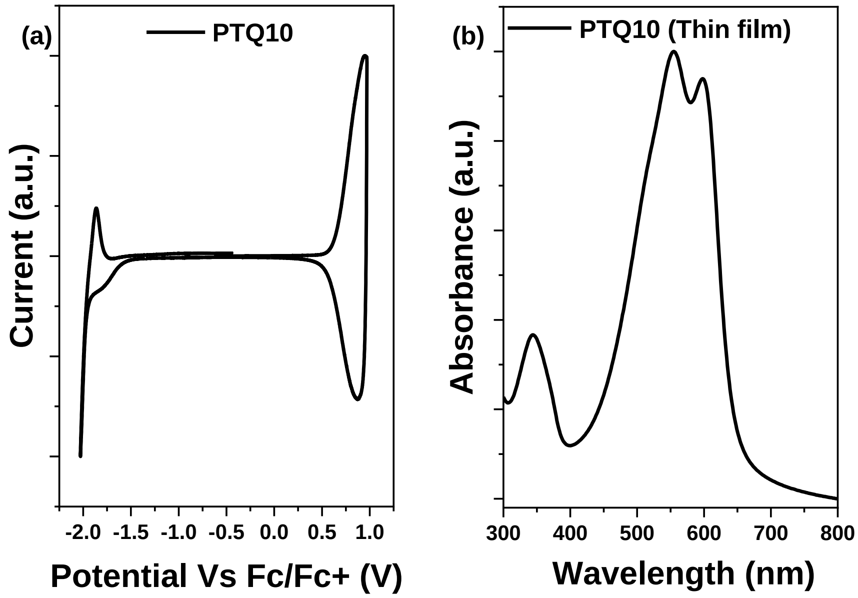

Figure S8. (a) Normalized cyclic voltammogram and (b) normalized UV-vis absorption spectrum of **PTQ10**.

Table S4. Optical and electrochemical properties of **PTQ10**.

Polymer	$\lambda_{max}^{film\ a)}$ [nm]	$\lambda_{onset}^{film\ a)}$ [nm]	$E_g^{opt\ a)}$	$E_g^{ele\ b)}$ [eV]	$E_{HOMO}^{b,\ c)}$ [eV]	$E_{LUMO}^{b,\ d)}$ [eV]
PTQ10	554, 597	650	1.91	2.57	−5.76	−3.19

a) Determined from UV–vis absorption spectra of thin films processed from chloroform. b) Determined from cyclic voltammetry. c) $E_{HOMO} = -(E_{ox}^{onset} + 5.13)$ eV, d) $E_{LUMO} = -(E_{red}^{onset} + 5.13)$ eV.

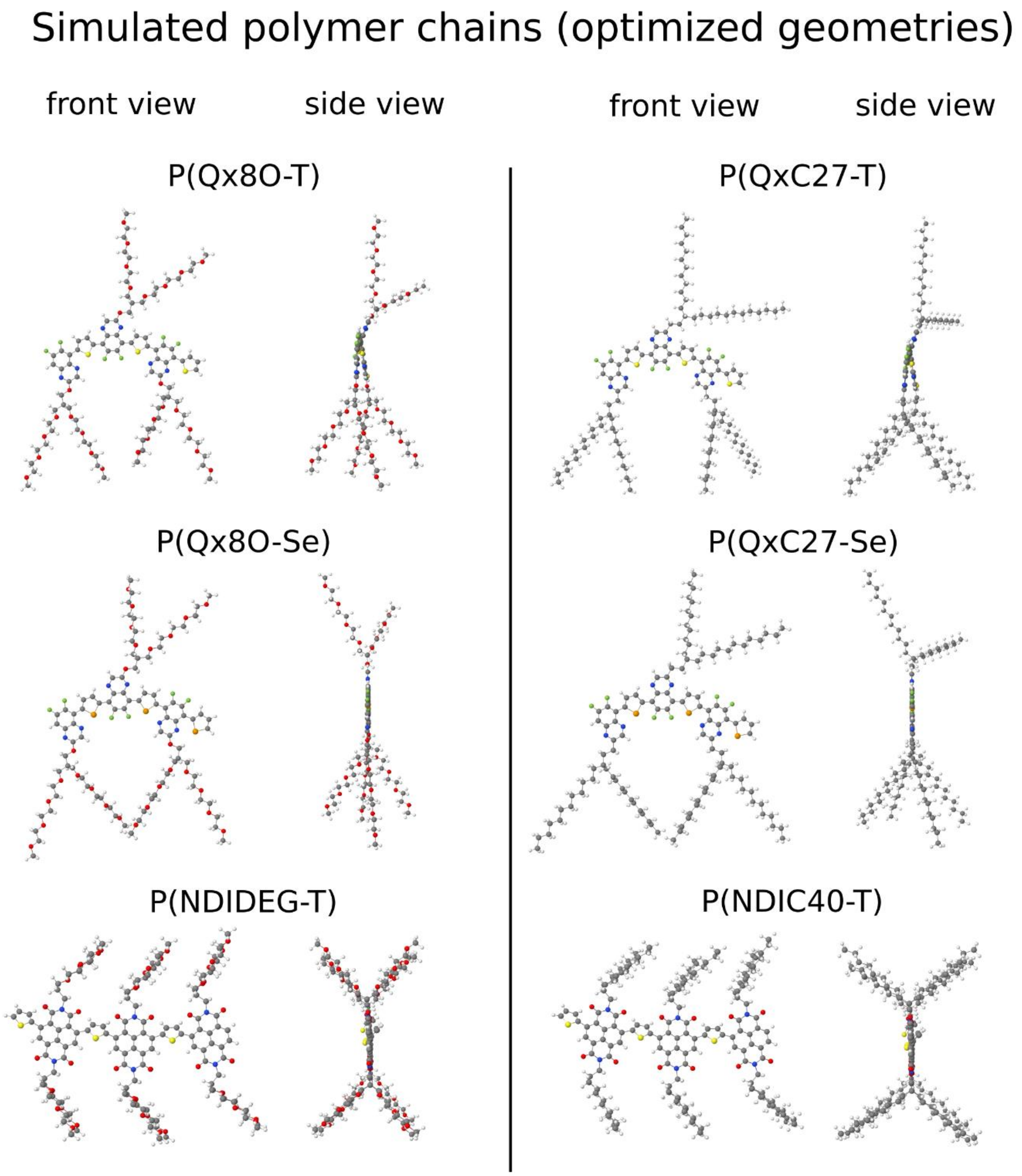

Figure S9. Ground State relaxed geometries (at HSE03/6-31G(d,p) theory level) of the trimeric donor and acceptor polymers.

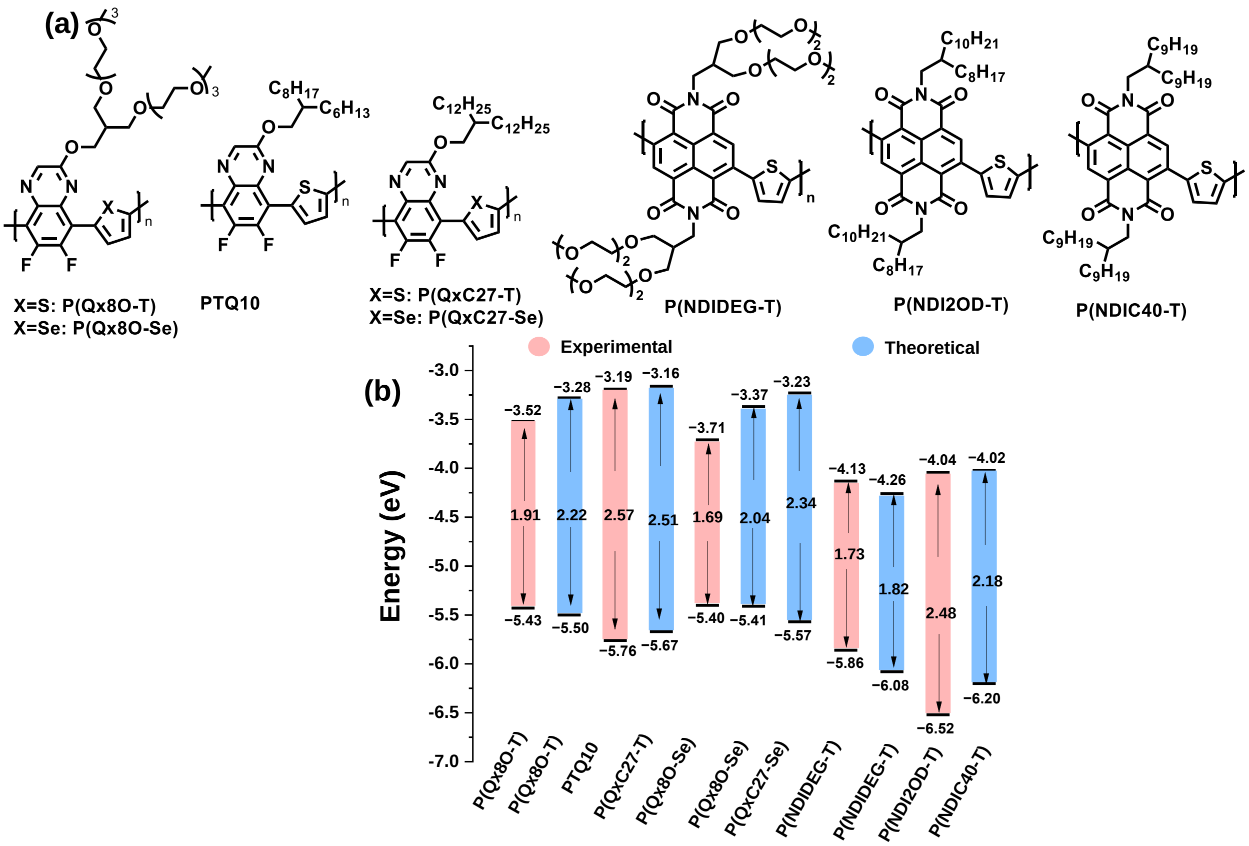

Figure S10. (a) Chemical structures of donor and acceptor polymers. (b) Energy levels of the donors and acceptor polymers, with the OEG and alkyl side chains, obtained from CV experiment and the Gibbs free energies of the oxidation and reduction reactions at the HSE03/6-311G(d,p) theory level. The HOMO and LUMO values of P(NDI2OD-T) was taken from the reported literature.[3]

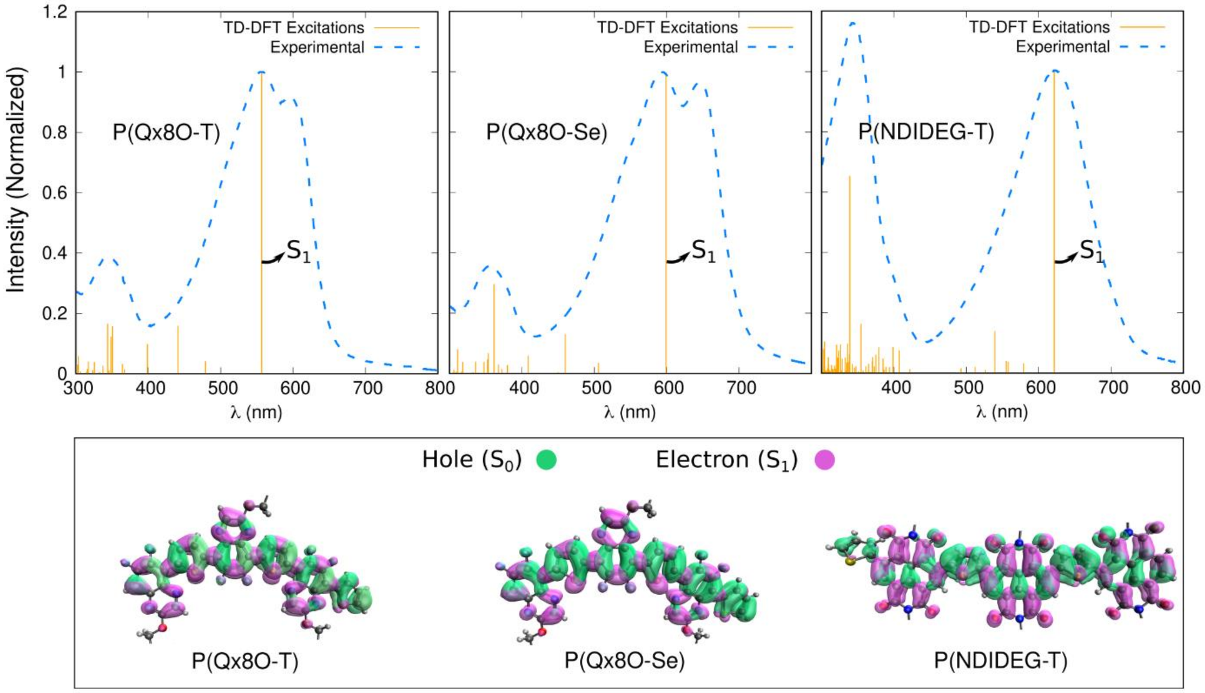

Figure S11. TD-DFT electronic excitations (orange vertical lines) of the donors and acceptor polymers in the UV-vis range. Experimental spectra plotted for comparison. Electron/hole picture of the first electronic transition (S_1) plotted with an isodensity value of 0.04 electrons/Å^3.

Fabrication of aq-APSC Devices

The aq-APSC devices were fabricated with the conventional structure, indium tin oxide (ITO)/ poly(3,4-ethylenedioxythiophene):polystyrene sulfonate (PEDOT:PSS) mixed with 0.15 vol% of (3-glycidoxypropyltrimethoxysilane) (GOPS)/active layer/poly[(9,9'-bis(3'-(*N*,*N*-dimethylamino)propyl)-2,7-fluorene)-*alt*-5,5'-bis(2,2'-thiophene)-2,6-naphthalene-1,4,5,8-tetracarboxylic-*N*,*N*'-di(2-ethylhexyl)imide] (PNDIT-F3N-Br)/silver (Ag). First, the glass/ITO substrates were washed by sonication in acetone, deionized water, and isopropyl alcohol, then dried in an oven for 30 min at 80°C. Next, the PEDOT:PSS solution (Clevios PVP AI4083) with 0.15 vol% of GOPS (for crosslinking the PEDOT:PSS layer to protect it from the aqueous solution of the active layer deposited above) was spin-coated onto the substrate at 3000 rpm for 40 s. After spin-coating, the PEDOT:PSS layer was thermally annealed at 150 °C under ambient conditions for 30 min. The quinoxaline-based polymer donors were blended with **P(NDIDEG-T)** polymer acceptor with an optimized donor:acceptor ratio of 2:1 (*w/w*) with a total concentration of 11.5 mg mL^{-1} at 90 °C for more than 30 min. Then, the active layer solution was spin-coated on top of the PEDOT:PSS layer at 1500 rpm for 40 s in ambient conditions. The film thickness of the resulting active layer was determined to be 80−90 nm. The devices were dried in a high-vacuum chamber ($<10^{-6}$ Torr) for more than 1 h. The PNDIT-F3N-Br electron transporting polymer was dissolved in methanol (1 mg mL^{-1}) and spin-coated on top of the active layer for 40 s at 2500 rpm under ambient conditions. The Ag electrode (120 nm) was deposited in a thermal evaporation chamber at high vacuum ($<10^{-6}$ Torr). Lastly, the devices were annealed in a vacuum annealing chamber at 100 ℃ for 2 min. The photoactive area of the devices was 0.042 cm^2 measured by optical microscopy.

The current density–voltage (*J*−*V*) characteristics of the aq-APSCs were obtained in ambient conditions under AM 1.5G solar irradiation (100 mW cm^{-2}, solar simulator: K201 LAB55, McScience). This solar simulator is certified to Class AAA for ASTM standards. The light intensity of the solar simulator was calibrated by using a standard silicon reference cell (K801S-K302, McScience) right before the measurements. The *J*–*V* properties were recorded with a Keithley 2400 SMU. The spectral measurement system (K3100 IQX, McScience Inc.) was used to collect the external quantum efficiency (EQE) data under dark conditions. The monochromatic light from a Xenon arc lamp at 300 W was filtered by a monochromator (Newport) and an optical

chopper (MC 2000 Thor labs). The calculated J_{sc} was determined by integrating the product of the EQE and the AM 1.5G solar spectrum.

Note 2. **Conformational preferences of thiophene and selenophene rings relative to acceptor units**

When the sulfur/selenium atom was turned toward the nitrogen atom of the Qx-moiety, lower electronic energy *ca* 1.15/2.47 kcal mol^{-1} was observed in a single repeating unit. This conformational preference might be mainly due to the preference of the S and Se-atoms softer electron donor in this case N-atoms in the Qx-ring. This argument also supported by the higher stabilization energy of the softer Se compared to S-atom. Another possible explanation for this could be the ring strain. The relative geometric location of the N-atom in the Qx-ring might not accept extended ring (6-membered) non-covalent interaction with H-atom. However, it is important to note that the interpolymer interactions between different chains in a polymeric bulk can stabilize other conformations, and therefore, require a more in-depth study. In the case of the acceptor, the presence of minima was observed solely when the thiophene hydrogens were directed towards the diimide's oxygen.

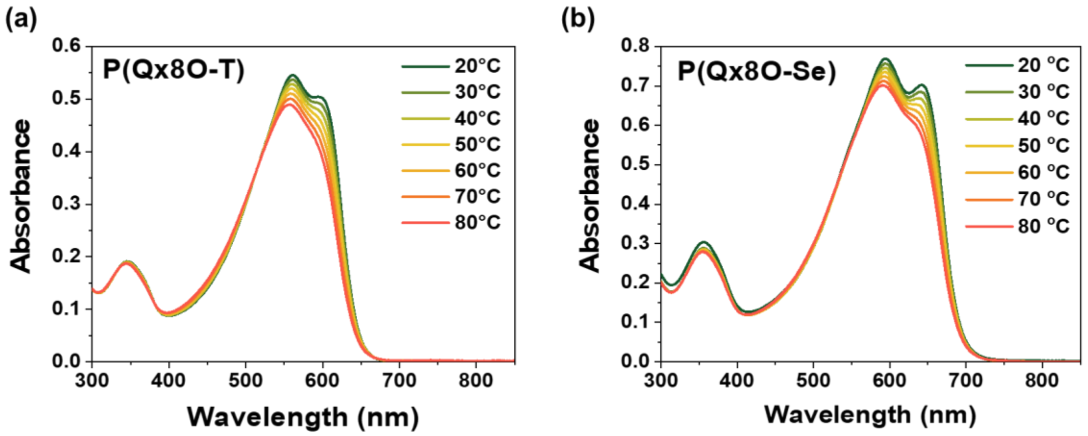

Figure S12. Temperature-dependent UV–vis absorption spectra of (a) **P(Qx8O-T)** and (b) **P(Qx8O-Se)** in water/ethanol (15:85 v/v) mixtures (0.02 mg mL^{-1}).

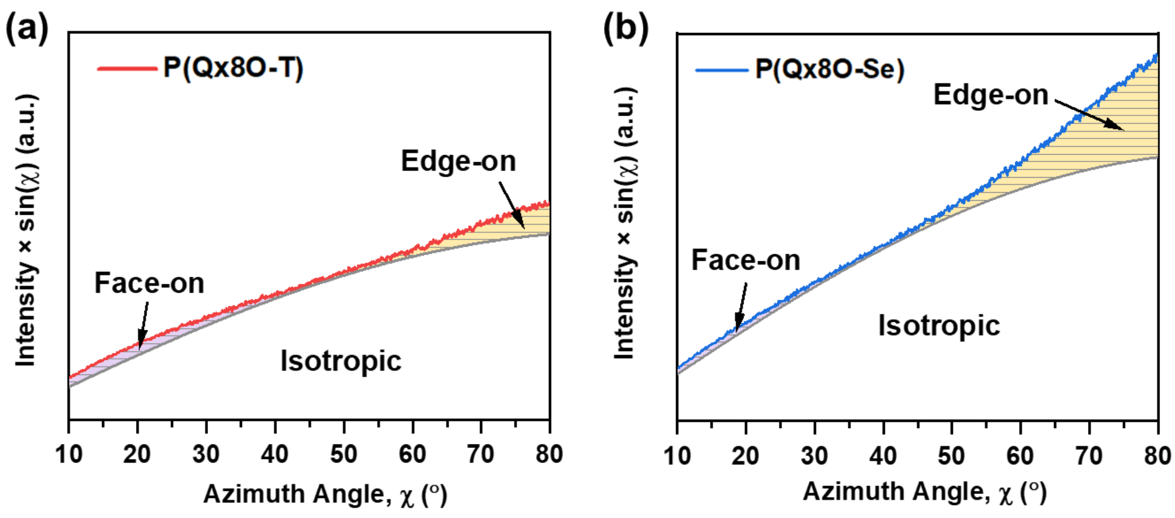

Figure S13. Pole figures obtained at (010) scattering peaks of (a) **P(Qx8O-T**) and (b) **P(Qx8O-Se**) films. The peak intensities were multiplied by a geometric factor, sin(χ).

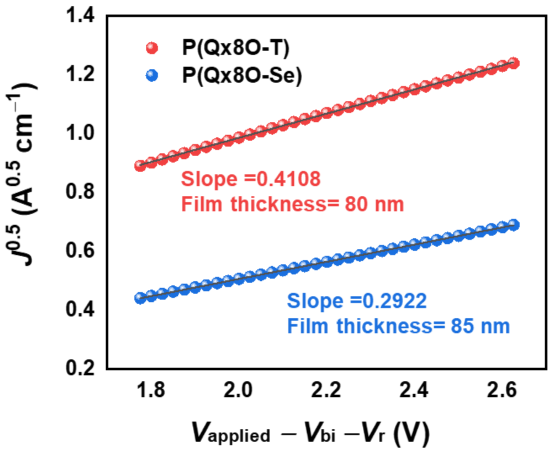

Figure S14. SCLC curves of pristine (a) **P(Qx8O-T)** and (b) **P(Qx8O-Se)** films.

Table S5. Photovoltaic performance of **P(Qx8O-T)**:**P(NDIDEG-T)** (donor:acceptor = 2:1 w/w) in various processing solvents.

Processing solvent	V_{OC} [V]	J_{SC} [mA cm^{-2}]	FF	PCE$_{avg}$ [a] (PCE$_{max}$) [%]
Chloroform	0.81	4.11	0.54	1.79 (1.88)
Acetone	0.70	5.13	0.44	1.58 (1.79)
Water/BuOH[b] (15:85 v/v)	0.73	2.55	0.50	0.93 (1.07)
Water/IPA[c] (15:85 v/v)	0.72	0.44	0.20	0.06 (0.08)
Water/EtOH [d] (5:95 v/v)	0.72	4.17	0.49	1.46 (1.50)
Water/EtOH [d] (15:85 v/v)	0.77	4.82	0.55	2.02 (2.27)
Water/EtOH [d] (25:75 v/v)	0.72	3.72	0.52	1.39 (1.48)
Water/EtOH [d] (35:65 v/v)	0.69	3.69	0.49	1.24 (1.29)
Ethyl acetate	0.75	4.42	0.42	1.41 1.55)

[a] Averaged from at least 3 devices. [b] Butanol. [c] Isopropyl alcohol. [d] Ethanol.

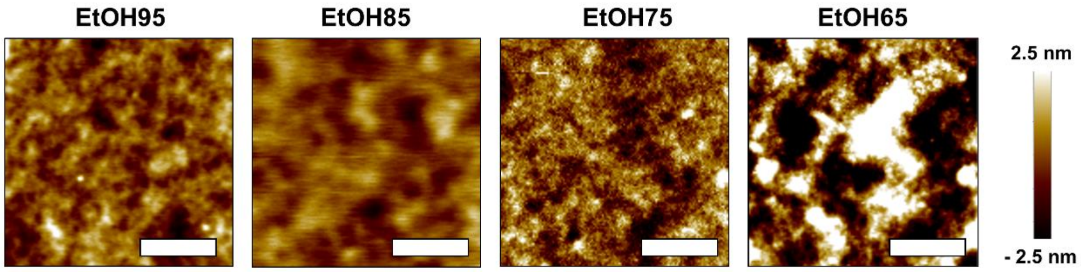

Figure S15. AFM height images of the **P(Qx8O-T)**:**P(NDIDEG-T)** blend films depending on the H_2O:EtOH volume ratio in EtOH95 (H_2O:EtOH, 5:95 (v/v)), EtOH85 (H_2O:EtOH, 15:85 (v/v)), ETOH75 (H_2O:EtOH, 25:75 (*v/v*)), and EtOH65 (H_2O:EtOH, 35:65 (v/v)) (scale bars: 1 μm).

Table S6. Summary of OSC performances of devices processed from water/ethanol (15:85 v/v).

D:A type	Active layer	V_{OC} [V]	J_{SC} [mA cm^{-2}]	FF	PCE$_{avg}$ (PCE$_{max}$) [%]	Ref.
**Polymer:Fullerene-Based Solar Cells**	PPDT2FBT-A:$PC_{61}BO_{15}$	0.73	4.06	0.49	1.39 (1.45)	[16]
	PPDT2FBT-A:$PC_{71}BO_{15}$	0.75	6.23	0.54	2.32 (2.51)	[16]
	PPDT2FBT-A:$PC_{61}BO_{12}$	0.76	5.08	0.53	1.86 (2.05)	[17]
	PFO4:$PC_{61}BO_{12}$	0.66	5.12	0.61	1.98 (2.06)	[18]
	PFO3:$PC_{61}BO_{12}$	0.72	6.50	0.65	2.92 (3.03)	[18]
**All-Polymer Solar Cells**	PPDT2FBT-A:P(NDIDEG-T)	0.66	6.43	0.44	1.89 (2.15)	[19]
	PPDT2FBT-A:P(NDITEG-T)	0.68	4.11	0.43	1.22 (1.43)	[19]
	PPDT2FBT-A:P(NDITEG-T2)	0.71	4.53	0.43	1.38 (1.56)	[19]
	**P(Qx8O-T):P(NDIDEG-T)**	**0.77**	**4.82**	**0.55**	**2.02 (2.27)**	***This work***
	**P(Qx8O-Se):P(NDIDEG-T)**	**0.70**	**4.46**	**0.55**	**1.72 (1.86)**	***This work***

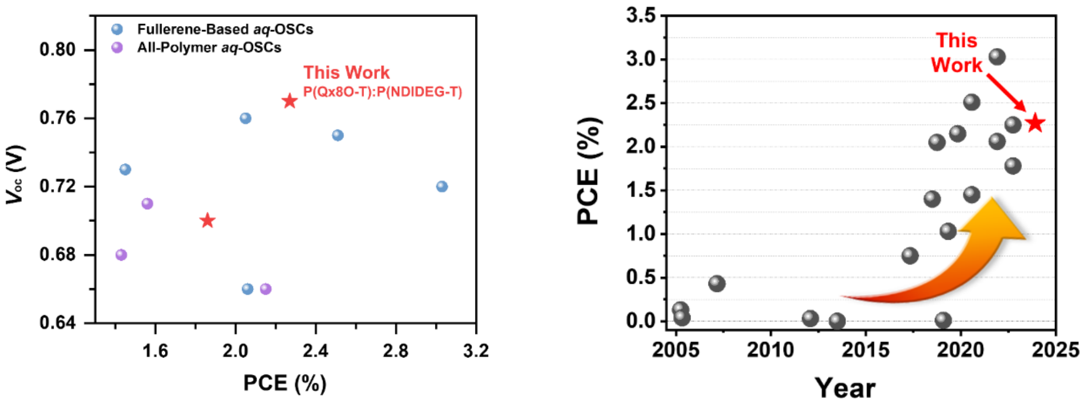

Figure S16. A plot of V_{OC} *vs* PCE of aq-OSCs (left) and performance of aq-OSCs reported to date.

Table S7. SCLC hole or electron mobilities of blend films and **P(NDIDEG-T)** pristine film.

Sample	μ_h [cm^2 V^{-1} s^{-1}]$^{a)}$	μ_e [cm^2 V^{-1} s^{-1}]$^{a)}$
**P(Qx8O-T)**:**P(NDIDEG-T)**	$(7.4 \pm 1.0) \times 10^{-5}$	$(8.6 \pm 1.4) \times 10^{-6}$
**P(Qx8O-Se)**:**P(NDIDEG-T)**	$(3.7 \pm 0.8) \times 10^{-5}$	$(5.8 \pm 0.6) \times 10^{-6}$
**P(NDIDEG-T)**	-	$(4.1 \pm 0.4) \times 10^{-5}$

a) Averaged from 5 SCLC devices for each system.

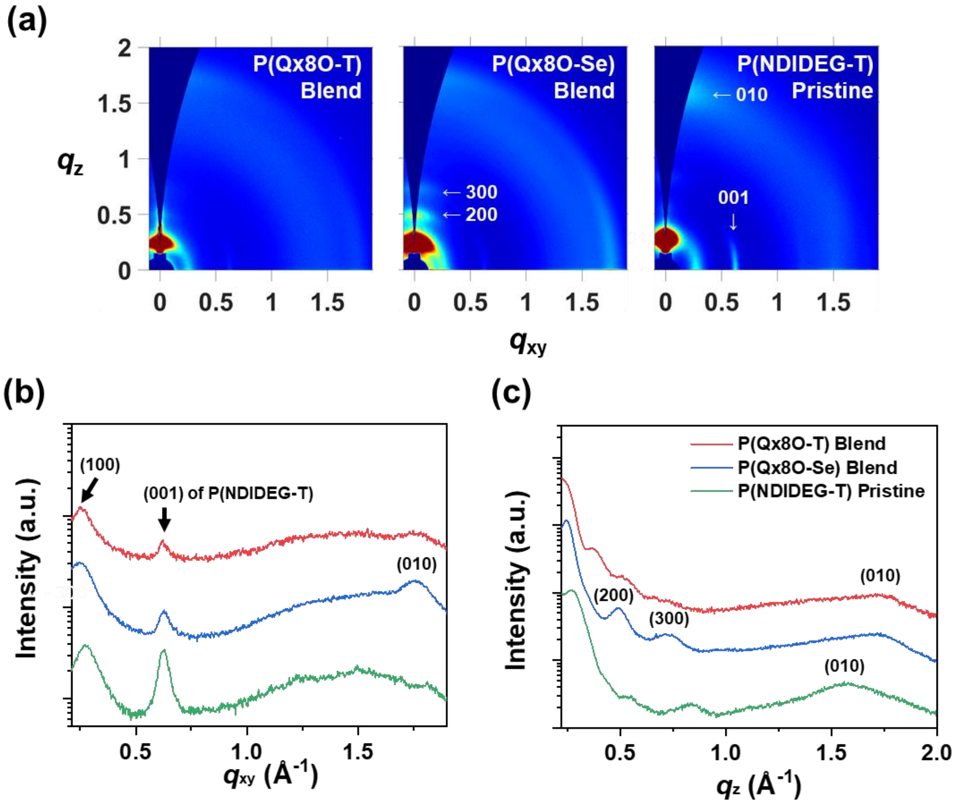

Figure S17. (a) 2D GIWAXS scattering patterns of **P(Qx8O-T)**:**P(NDIDEG-T)** and **P(Qx8O-Se)**:**P(NDIDEG-T)** blend films and **P(NDIDEG-T)** pristine film. Their line-cut profiles in the (b) IP and (c) OOP directions.

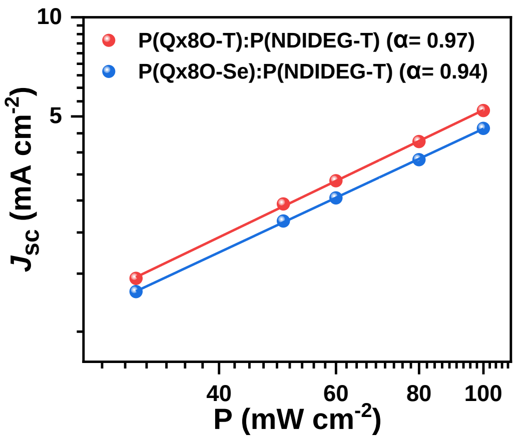

Figure S18. *P*-dependent J_{sc} of **P(Qx8O-T)**:**P(NDIDEG-T)** and **P(Qx8O-Se)**:**P(NDIDEG-T)** devices.

Table S8. Domain size and relative domain purity values of **P(Qx8O-T)**:**P(NDIDEG-T)** and **P(Qx8O-Se)**:**P(NDIDEG-T)** calculated from the RSoXS profiles.

Blend Film	Domain Size (nm)	Relative Domain Purity
**P(Qx8O-T)**:**P(NDIDEG-T)**	69	0.92
**P(Qx8O-Se)**:**P(NDIDEG-T)**	73	1.00

Table S9. Photovoltaic performance of PTQ10:P(NDI2OD-T)- and **P(Qx8O-T)**:**P(NDIDEG-T)**-based devices before and after immersing the devices underwater.

Active Layer [a]	Immersing Underwater	V_{oc} [V]	J_{sc} [mA cm^{-2}]	FF	PCE$_{avg}$ (PCE$_{max}$) [%]
PTQ10: P(NDI2OD-T) [b]	Before	0.71±0.03	4.80±0.20	0.32±0.01	1.08±0.13 (1.29)
	After	0.61±0.06	4.09±0.37	0.29±0.01	0.74±0.17 (0.94)
**P(Qx8O-T)**: **P(NDIDEG-T)** [c]	Before	0.75±0.00	5.59±0.17	0.44±0.02	1.85±0.12 (1.99)
	After	0.72±0.02	4.52±0.20	0.42±0.01	1.38±0.11 (1.53)

[a] To solely observe the effects of the processing solvents and active materials, we unified the other fabrication conditions (*i.e.*, device structure: ITO/PEDOT:PSS + 0.15 vol% GOPS/active layer/PNDIT-F3N-Br/Ag, donor:acceptor ratio = 2:1 (w/w), total concentration 11.5 mg mL^{-1}). The thickness of the active layers was 80–90 nm. [b] Processed with chloroform. M_n of PTQ10 = 47 kg mol^{-1}; M_n of P(NDI2OD-T) = 37 kg mol^{-1}. [c] Processed with water:ethanol (15:85 v/v).

References

1. Lu, T.; Chen, F., Quantitative analysis of molecular surface based on improved Marching Tetrahedra algorithm. *J. Mol. Graphics Modell.* **2012,** *38*, 314-323.
2. Lu, T.; Chen, F., Multiwfn: A multifunctional wavefunction analyzer. *J. Comput. Chem.* **2012,** *33* (5), 580-592.
3. Li, Z.; Zhang, W.; Xu, X.; Genene, Z.; Di Carlo Rasi, D.; Mammo, W.; Yartsev, A.; Andersson, M. R.; Janssen, R. A. J.; Wang, E., High-Performance and Stable All-Polymer Solar Cells Using Donor and Acceptor Polymers with Complementary Absorption. *Adv. Energy Mater.* **2017,** *7* (14), 1602722.
4. Chen, X.; Zhang, Z.; Ding, Z.; Liu, J.; Wang, L., Diketopyrrolopyrrole-based Conjugated Polymers Bearing Branched Oligo(Ethylene Glycol) Side Chains for Photovoltaic Devices. *Angew. Chem. Int. Ed.* **2016,** *55*, 10376-10380.
5. Wu, J.; You, L.; Lan, L.; Lee, H. J.; Chaudhry, S. T.; Li, R.; Cheng, J.-X.; Mei, J., Semiconducting Polymer Nanoparticles for Centimeters-Deep Photoacoustic Imaging in the Second Near-Infrared Window. *Adv. Mater.* **2017,** *29* (41), 1703403.
6. Zhu, S.; Zhang, J.; Vegesna, G.; Luo, F.-T.; Green, S. A.; Liu, H., Highly Water-Soluble Neutral BODIPY Dyes with Controllable Fluorescence Quantum Yields. *Org. Lett.* **2011,** *13* (3), 438-441.
7. Sun, C.; Pan, F.; Bin, H.; Zhang, J.; Xue, L.; Qiu, B.; Wei, Z.; Zhang, Z.-G.; Li, Y., A low cost and high performance polymer donor material for polymer solar cells. *Nat. Commun.* **2018,** *9* (1), 743.

8. Rech, J. J.; Neu, J.; Qin, Y.; Samson, S.; Shanahan, J.; Josey III, R. F.; Ade, H.; You, W., Designing Simple Conjugated Polymers for Scalable and Efficient Organic Solar Cells. *ChemSusChem* **2021,** *14* (17), 3561-3568.

9. Heyd, J.; Scuseria, G. E.; Ernzerhof, M., Hybrid functionals based on a screened Coulomb potential. *J. Chem. Phys.* **2003,** *118* (18), 8207-8215.

10. Krishnan, R.; Binkley, J. S.; Seeger, R.; Pople, J. A., Self-consistent molecular orbital methods. XX. A basis set for correlated wave functions. *J. Chem. Phys.* **1980,** *72* (1), 650-654.

11. Marenich, A. V.; Cramer, C. J.; Truhlar, D. G., Universal Solvation Model Based on Solute Electron Density and on a Continuum Model of the Solvent Defined by the Bulk Dielectric Constant and Atomic Surface Tensions. *J. Phys. Chem. B* **2009,** *113* (18), 6378-6396.

12. Donaghey, J. E.; Armin, A.; Burn, P. L.; Meredith, P., Dielectric constant enhancement of non-fullerene acceptors via side-chain modification. *Chem. Commun.* **2015,** *51* (74), 14115-14118.

13. Fu, Z.; Zhang, X.; Zhang, H.; Li, Y.; Zhou, H.; Zhang, Y., On the Understandings of Dielectric Constant and Its Impacts on the Photovoltaic Efficiency in Organic Solar Cells. *Chin. J. Chem* . **2021,** *39* (2), 381-390.

14. Rousseva, S.; Besten, H. d.; van Kooij, F. S.; Doting, E. L.; Doumon, N. Y.; Douvogianni, E.; Anton Koster, L. J.; Hummelen, J. C., Reaching a Double-Digit Dielectric Constant with Fullerene Derivatives. *J. Phys. Chem. C* **2020,** *124* (16), 8633-8638.

15. Frisch, M. J.; Trucks, G. W.; Schlegel, H. B.; Scuseria, G. E.; Robb, M. A.; Cheeseman, J. R.; Scalmani, G.; Barone, V.; Petersson, G. A.; Nakatsuji, H.; Li, X.; Caricato, M.; Marenich, A. V.; Bloino, J.; Janesko, B. G.; Gomperts, R.; Mennucci, B.; Hratchian, H. P.; Ortiz, J. V.; Izmaylov, A. F.; Sonnenberg, J. L.; Williams; Ding, F.; Lipparini, F.; Egidi,

F.; Goings, J.; Peng, B.; Petrone, A.; Henderson, T.; Ranasinghe, D.; Zakrzewski, V. G.; Gao, J.; Rega, N.; Zheng, G.; Liang, W.; Hada, M.; Ehara, M.; Toyota, K.; Fukuda, R.; Hasegawa, J.; Ishida, M.; Nakajima, T.; Honda, Y.; Kitao, O.; Nakai, H.; Vreven, T.; Throssell, K.; Montgomery Jr., J. A.; Peralta, J. E.; Ogliaro, F.; Bearpark, M. J.; Heyd, J. J.; Brothers, E. N.; Kudin, K. N.; Staroverov, V. N.; Keith, T. A.; Kobayashi, R.; Normand, J.; Raghavachari, K.; Rendell, A. P.; Burant, J. C.; Iyengar, S. S.; Tomasi, J.; Cossi, M.; Millam, J. M.; Klene, M.; Adamo, C.; Cammi, R.; Ochterski, J. W.; Martin, R. L.; Morokuma, K.; Farkas, O.; Foresman, J. B.; Fox, D. J. *Gaussian 16 Rev. A.03*, Wallingford, CT, 2016.

16. Kim, C.; Kang, H.; Choi, N.; Lee, S.; Kim, Y.; Kim, J.; Wu, Z.; Woo, H. Y.; Kim, B. J., C70-based aqueous-soluble fullerene for the water composition-tolerant performance of eco-friendly polymer solar cells. *J. Mater. Chem. C* **2020,** *8* (43), 15224-15233.
17. Lee, C.; Lee, H. R.; Choi, J.; Kim, Y.; Nguyen, T. L.; Lee, W.; Gautam, B.; Liu, X.; Zhang, K.; Huang, F.; Oh, J. H.; Woo, H. Y.; Kim, B. J., Efficient and Air-Stable Aqueous-Processed Organic Solar Cells and Transistors: Impact of Water Addition on Processability and Thin-Film Morphologies of Electroactive Materials. *Adv. Energy Mater.* **2018,** *8* (34), 1802674.
18. Shang, L.; Qu, S.; Deng, Y.; Gao, Y.; Yue, G.; He, S.; Wang, Z.; Wang, Z.; Tan, F., Simple furan-based polymers with the self-healing function enable efficient eco-friendly organic solar cells with high stability. *J. Mater. Chem. C* **2022,** *10* (2), 506-516.
19. Lee, S.; Kim, Y.; Wu, Z.; Lee, C.; Oh, S. J.; Luan, N. T.; Lee, J.; Jeong, D.; Zhang, K.; Huang, F.; Kim, T.-S.; Woo, H. Y.; Kim, B. J., Aqueous-Soluble Naphthalene Diimide-Based

Polymer Acceptors for Efficient and Air-Stable All-Polymer Solar Cells. *ACS Appl. Mater. Interfaces* **2019,** *11* (48), 45038-45047.
